# CD5L-associated gene analyses highlight the dysregulations, prognostic effects, immune associations, and drug-sensitivity predicative potentials of LCAT and CDC20 in hepatocellular carcinoma

**DOI:** 10.1186/s12935-022-02820-7

**Published:** 2022-12-09

**Authors:** Xiuzhi Zhang, Xiaoli Liu, Keke Zhu, Xue Zhang, Ningning Li, Tao Sun, Shasha Fan, Liping Dai, Jinzhong Zhang

**Affiliations:** 1 Department of Pathology, Henan Medical College, Zhengzhou, China; 2 Laboratory Department, Henan Provincial People’s Hospital, Zhengzhou, China; 3 Henan Institute of Medical and Pharmaceutical Sciences, Zhengzhou University, Zhengzhou, China; 4 Oncology Department, The First Affiliated Hospital of Hunan Normal University, Hunan Provincial People’s Hospital, Changsha, China; 5 Key Laboratory of Study and Discovery of Small Targeted Molecules of Hunan Province, Hunan Normal University, Changsha, China

**Keywords:** CD5L, LCAT, CDC20, Hepatocellular carcinoma (HCC), Lipid metabolism, Immune response, Diagnosis, Prognosis

## Abstract

**Background:**

The dysregulation of CD5L has been reported in hepatocellular carcinoma (HCC). However, its functions in HCC were controversial. In this study, we aimed to identify CD5L-associated pathways and markers and explore their values in HCC diagnosis, prognosis and treatment.

**Methods:**

HCC datasets with gene expression profiles and clinical data in TCGA and ICGC were downloaded. The immune/stroma cell infiltrations were estimated with xCell. CD5L-associated pathways and CD5L-associated genes (CD5L-AGs) were identified with gene expression comparisons and gene set enrichment analysis (GSEA). Cox regression, Kaplan–Meier survival analysis, and least absolute shrinkage and selection operator (LASSO) regression analysis were performed. The correlations of the key genes with immune/stroma infiltrations, immunoregulators, and anti-cancer drug sensitivities in HCC were investigated. At protein level, the key genes dysregulations, their correlations and prognostic values were validated in clinical proteomic tumor analysis consortium (CPTAC) database. Serum CD5L and LCAT activity in 50 HCC and 30 normal samples were evaluated and compared. The correlations of serum LCAT activity with alpha-fetoprotein (AFP), albumin (ALB) and high-density lipoprotein (HDL) in HCC were also investigated.

**Results:**

Through systemic analyses, 14 CD5L-associated biological pathways, 256 CD5L-AGs and 28 CD5L-associated prognostic and diagnostic genes (CD5L-APDGs) were identified. A risk model consisting of LCAT and CDC20 was constructed for HCC overall survival (OS), which could discriminate HCC OS status effectively in both the training and the validation sets. CD5L, LCAT and CDC20 were shown to be significantly correlated with immune/stroma cell infiltrations, immunoregulators and 31 anti-cancer drug sensitivities in HCC. At protein level, the dysregulations of CD5L, LCAT and CDC20 were confirmed. LCAT and CDC20 were shown to be significantly correlated with proliferation marker MKI67. In serum, no significance of CD5L was shown. However, the lower activity of LCAT in HCC serum was obvious, as well as its significant positive correlations ALB and HDL concentrations.

**Conclusions:**

CD5L, LCAT and CDC20 were dysregulated in HCC both at mRNA and protein levels. The LCAT-CDC20 signature might be new predicator for HCC OS. The associations of the three genes with HCC microenvironment and anti-cancer drug sensitivities would provide new clues for HCC immunotherapy and chemotherapy.

**Supplementary Information:**

The online version contains supplementary material available at 10.1186/s12935-022-02820-7.

## Background

As one of the most prevalent malignancies worldwide, primary liver cancer accounts for 4.7% of the cancer incidence and 8.3% of the cancer mortality in the year 2020 [1]. For liver cancer, approximately 80% of the cases are hepatocellular carcinoma (HCC) [2] and the 5-year overall survival (OS) rate is below 20% [3]. The dismal prognosis of HCC is mainly due to its late diagnosis, tumor recurrence and drug-resistance. To improve HCC prognosis, it is very important to develop effective markers for its early diagnosis and find sensitive therapeutic targets for its treatment.

As two hallmarks of cancer, metabolism dysregulation and immune evasion are common in cancerous diseases [4, 5]. In HCC, many metabolic genes were shown to be involved in its development and progression [6–8]. In a recent study, fructose-1,6-bisphosphatase inhibition was found to be associated be HCC growth and metastasis [9]. A signature of six metabolic genes including G6PD, AKR1B15, HMMR, CSPG5, ELOVL3 and FABP6 was shown to be predictive for HCC prognosis [10]. The associations between metabolism dysregulation and immune response were presented in many studies [11]. In HCC, reprogramming of lipid metabolism was confirmed to be involved in HCC development and immunoregulation [12–14]. The in-depth study of the key genes implicated in lipid metabolism and immune response would be helpful for understanding HCC occurrence and progression.

CD5-like molecule (CD5L), also known as apoptosis inhibitor of macrophages (AIM), has been reported to have multiple functions in lipid metabolism [15] and inflammatory processes [15–17]. In recently years, its involvement in cancerous disease was also demonstrated in several malignancies. Its overexpression in alveolar type II epithelial cells was found to be associated with the occurrence of lung adenocarcinoma [18]. In prostate cancer, the serum CD5L was shown to be higher than that of benign prostatic hyperplasia [19]. In contrast to its tumor-promoting potential, other studies also demonstrated its anti-tumor activities. It was reported that HCC could be induced through high-fat diet in CD5L-lacking mice while not in the wild-type ones [20]. Its anti-HCC activity in mice was also shown through its prevention of hepatocellular carcinoma with administration of CD5L [21]. In a clinical study, higher serum CD5L protein was also demonstrated to be associated with the good response of HCC patients to sorafenib treatment [22]. In our previous study, at gene expression level, CD5L was shown to be decreased in HCC samples and its favorable prognostic effects on HCC survival were presented [23]. However, in another study, CD5L was demonstrated to be higher expressed in HCC (n = 60) than the normal controls (n = 34) through immunochemistry analysis and its HCC-promoting activity was shown in HCC cell lines [24]. These seemingly opposite results indicated the complexity and diversity of CD5L functions in HCC to some extent.

Liver has crucial roles in lipid metabolism and immunoregulation [25] which are also tightly associated with CD5L function [16]. Thus, it is necessary to do an in-depth and systemic study of CD5L in HCC to uncover its potential functions. In this study, we investigated CD5L-associated genes and pathways with HCC datasets from The Cancer Genome Atlas (TCGA) and International Cancer Genome Consortium (ICGC). The dysregulations and prognostic effects of the CD5L-correlated genes were evaluated. A risk model was constructed for HCC OS and two CD5L-associated genes were identified as key genes for further analysis. Their associations with HCC microenvironment, immunoregulatory gene expressions and anti-cancer drug sensitivities were shown in HCC. At protein level, their dysregulations were confirmed and their prognostic effects were also shown. Furthermore, the lower activity of serum LCAT in HCC and its positive correlations with ALB and HDL concentrations were uncovered. These results might provide new clues for the functions of CD5L in HCC, new markers for its early diagnosis and prognostic predication of HCC, and new targets for its treatment.

## Materials and methods

### Data collection and processing

RNA-seq data of HCC tumors and their paired normal liver tissue controls in LIHC (liver hepatocellular carcinoma, called HCC in this study) datasets with the corresponding clinical information were downloaded from Genomic Data Commons (GDC) data portal (https://portal.gdc.cancer.gov/, TCGA-HCC dataset) and International Cancer Genome Consortium (ICGC) (https://dcc.icgc.org/, ICGC-HCC dataset). There were 371 and 223 primary HCC tumors in TCGA-HCC dataset and ICGC-HCC dataset, respectively. Their paired normal controls were 50 and 202, respectively. The clinical characteristics of the patients were shown in Additional file 1: Table S1. For further analyses, gene expression normalization was performed and transcripts per million (TPM) was used.

### Gene Set Enrichment Analysis (GSEA) of CD5L and CD5L-associated gene (CD5L-AG) identification in HCC

To explore the potential roles of CD5L in HCC development and progression, the primary HCC samples in each HCC dataset were divided into two groups (CD5L^high^ group and CD5L^low^ group) with the median expression of CD5L for comparisons. Gene expression differences between the two groups were evaluated with “limma” package [26] in R. With the gene expression changes [log_2_(fold change), LogFC], GSEA was performed with “GSEABase” package in R (https://rdrr.io/bioc/GSEABase) and the hallmark gene sets (n = 50) in the Molecular Signatures Database (MSigDB) (https://www.gsea-msigdb.org/gsea/msigdb/genesets.jsp?collection=H) were included. The related gene sets with Benjamini and Hochberg (BH) adjusted *p* value (*p*.adj) < 0.05 consistently in the two datasets were considered significant. The genes in the significant gene sets and differentially expressed between CD5L^high^ and CD5L^low^ groups were considered as CD5L-associated genes (CD5L-AGs).

### Evaluation of the prognostic effects and dysregulations of CD5L-AGs in HCC

The age-gender-stage-corrected prognostic effects of CD5L-AGs were investigated in TCGA-HCC and ICGC-HCC datasets with “ezcox” package in R [27]. For the analysis, *p* < 0.05 was considered significant and only the CD5L-AGs with consistent prognostic effects on HCC OS were considered as CD5L-associated prognostic genes (CD5L-APGs). With “limma” package, gene expression comparisons between HCC tumors and normal liver tissues were evaluated and the expressional differences of the CD5L-APGs were extracted. The CD5L-APGs with most significant expressional dysregulation ( |logFC|> 1 and *p*.adj < 1e-10) in HCC tumors comparing with normal liver tissues in the two datasets were selected for further analyses and they were called CD5L-associated prognostic and diagnostic genes (CD5L-APDGs).

To find the most valuable CD5L-APDGs, least absolute shrinkage and selection operator (LASSO) Cox regression analysis was performed, and a risk model was constructed for HCC OS. The risk model was expressed as follows:

$$risk\,score= \sum_{i=1}^{n}coef\left(i\right)*exp(i)$$

where *n* represents the number of included genes, *coef(i)* denotes the coefficient of the gene *i*, and *exp(i)* is the expression level of gene *i*. The TCGA-HCC dataset and ICGC-HCC dataset were used as the training cohort and the validation cohort, respectively. To ensure the homogeneity of the LASSO Cox regression analysis in the two datasets, with “mosaic” package (https://cran.r-project.org/web/packages/mosaic/index.html), the gene expressions (TPMs) were further zscore normalized in R. Receiver Operating Characteristic (ROC) analysis was performed to present the prognostic power of the risk model and diagnostic power of the most significant CD5L-APDGs. For visualization of the correlations of the most significant CD5L-APDGs with CD5L and their prognostic effects, spearman correlation analysis and Kaplan–Meier survival analysis were performed with “ggplot2” package (https://cran.r-project.org/web/packages/ggplot2/index.html) and “survminer” package (https://cran.r-project.org/web/packages/survminer/index.html) in R. For the above analyses, *p* < 0.05 was considered statistically significant.

### Further insight of the associations of CD5L and the most significant CD5L-APDGs (LCAT and CDC20) with HCC microenvironment and immunoregulators

The relative abundance of 64 immune and stromal cells, the immune score, the stroma score, and the microenvironment score of tumor samples in TCGA-HCC and ICGC-HCC datasets were evaluated with xCell [28]. The differences of the cell infiltrations and the scores between HCC samples and liver controls were investigated. Their correlations with CD5L, LCAT, and CDC20 were evaluated. The age-gender-stage-corrected prognostic effects of the immune and stroma cells were investigated through Cox regression analysis with “ezcox” package [27] in R. Furthermore, a total of 91 immune regulators including 24 immunoinhibitors, 46 immunostimulators, and 21 major histocompatibility complex (MHC) related genes were downloaded from the Cancer Immunome Atlas (TCIA) (https://www.tcia.at) and the correlations of CD5L, LCAT and CDC20 with the immune regulators were estimated. Considering the regulatory functions of NF-κB pathway and the crucial effects of chemokines, and chemokine receptors in immune response[29–32], CD5L, LCAT, and CDC20 were also investigated for their correlations with five NF-KB-associated genes (NFKB1, NFKB2, REL, RELA, and RELB), 41 chemokines, and 18 chemokine receptors in TCGA-HCC and ICGC-HCC datasets. Wilcoxon test and spearman correlation analysis were used for comparisons and correlation estimation, respectively. For these analyses, *p* < 0.05 was considered statistically significant.

### Exploration of the associations of CD5L, LCAT and CDC20 with the sensitivity of HCC cell lines to present anti-cancer drugs

The relative expressions of CD5L, LCAT and CDC20 in HCC cell lines were downloaded from Cancer Cell Line Encyclopedia (CCLE) database. The pharmacologic profiles for 192 anti-cancer drugs across 809 cell lines were downloaded from Genomics of Drug Sensitivity in Cancer (GDSC) and the anti-cancer drugs with HCC cell lines were extracted. Spearman’s correlation analysis was also applied to investigate the associations between the three gene expressions and log-transformed half maximal inhibitory concentrations (LN_IC50s) of the drugs in the HCC cell lines. *P* < 0.05 was considered significant.

### Validation of CD5L, LCAT and CDC20 in HCC at protein level

The HCC dataset in Clinical Proteomic Tumor Analysis Consortium (CPTAC) was used for validation of the results above. The clinical characters of the samples were shown in Additional file 1: Table S2.

As clinical stage is very important for the gene expression comparisons, we estimated the T stage of the tumors based on the clinical data of the patients in CPTAC dataset and compared the T stage proportions of CPTAC dataset and the TCGA-HCC dataset. The protein expressions were also compared between normal liver samples and HCC samples of different T stages. The correlations of CD5L expression with LCAT and CDC20 expressions were evaluated with Spearman correlation analysis. The expressional differences of CD5L, LCAT and CDC20 proteins between HCC tumors and normal liver tissues were investigated with Wilcoxon tests. The prognostic and diagnostic effects of CD5L, LCAT and CDC20 at protein level were evaluated with Kaplan–Meier survival analysis and ROC curve analysis, respectively.

To investigate the associations of CD5L, LCAT and CDC20 with the tumor proliferation, their correlations with proliferation marker MKI67 [33, 34] were evaluated. Considering that AFP is the most widely used tumor marker in HCC [35], the correlations of the three proteins with AFP expression in HCC tissues (tissue AFP) and serum AFP levels were also investigated. ALB is produced by the liver and it is associated with liver function [36]. The associations of CD5L, LCAT and CDC20 with liver function in HCC tissues were evaluated through their Spearman’s correlations with tissue ALB and serum ALB levels.

The subcellular locations of CD5L, LCAT and CDC20 as well as their immunohistochemical staining in liver and HCC tissues were investigated via Human Protein Atlas (HPA, https://www.proteinatlas.org/). According to the human secretome data in HPA, CD5L and LCAT were secretory proteins while no transcript of CDC20 was predicted to be a secreted protein.

### Detection of serum CD5L and LCAT in HCC and normal controls

The serum levels of CD5L protein and LCAT activity in 50 HCC samples and 30 normal controls were evaluated with CD5L enzyme linked immunosorbent assay (ELISA) kit (RAB1347, Sigma-Adrich Ltd, USA) and LCAT activity assay kit (MAK107, Sigma-Adrich Ltd, USA). In addition, the concentrations of AFP (REF04491742190, Roche, Germany), ALB (F103T, MedicalSystem Ltd, China), and HDL (F203T, MedicalSystem Ltd, China) were also detected for further analyses. The work involving the serum specimens was reviewed and approved by the Ethics Committee of Henan People’s Hospital (approval number: 201948). All samples were collected with informed consent in accordance with the Declaration of Helsinki. The samples were obtained between February 2020 and August 2020 from Henan People’s Hospital (Zhengzhou, China) at the time of diagnosis before any therapy. The clinical features of the samples were shown in Table 1. The T stage proportions of these patients were compared with HCC patients in CPTAC dataset with Chi-square test. CD5L concentration and the relative activity of LCAT (the relative intensity of hydrolyzed substrate/the relative intensity of intact substrate) were measured in duplicate according to the manufacturer’s instructions. Wilcoxon test was used for the comparisons between HCC patients and normal controls. ROC analysis was performed to investigate the diagnostic potential of serum LCAT. Spearman correlation analysis was performed to investigate the associations of LCAT activity with AFP, ALB and HDL concentrations. For all the analysis, *p* < 0.05 was considered as significant.

**Table 1:** Clinical characteristics of HCC patients and normal controls for serum analyses

	HCC (n = 50)	Normal (n = 30)	*P*
Age (y)	32–75; median:53	34–77; median:54	0.724
Gender (male/female)	44/6	24/6	0.518
AFP (ng/ml)	7.3–121,000; median: 650.95	0.860–6.360; median: 3.295	9.428E-14^**^
ALB (g/l)	18.6–49.7; median: 36.90	41.5–51.3; median: 45.80	3.6E-11^**^
HDL (mmol/l)	0.360–1.600; median:1.025	0.660–1.850; median:1.325	0.000138^**^
Tumor stage (I/II /III /IV)	5/14/22/9		

^**^p < 0.01. Chi-square test and Wilcoxon test were used for comparisons and *p* < 0.05 was considered significant

### Investigation of the protein-chemical interactions of CD5L, LCAT and CDC20

To present the chemicals which might interact with them and have associations with HCC development, the protein-chemical interactions of CD5L, LCAT and CDC20 were investigated through NetworkAnalyst (https://www.networkanalyst.ca/) based on Comparative Toxicogenomics Database (CTD) (http://ctdbase.org/).

## Results

### GSEA of CD5L and CD5L-AGs in HCC

**Fig. 1:**
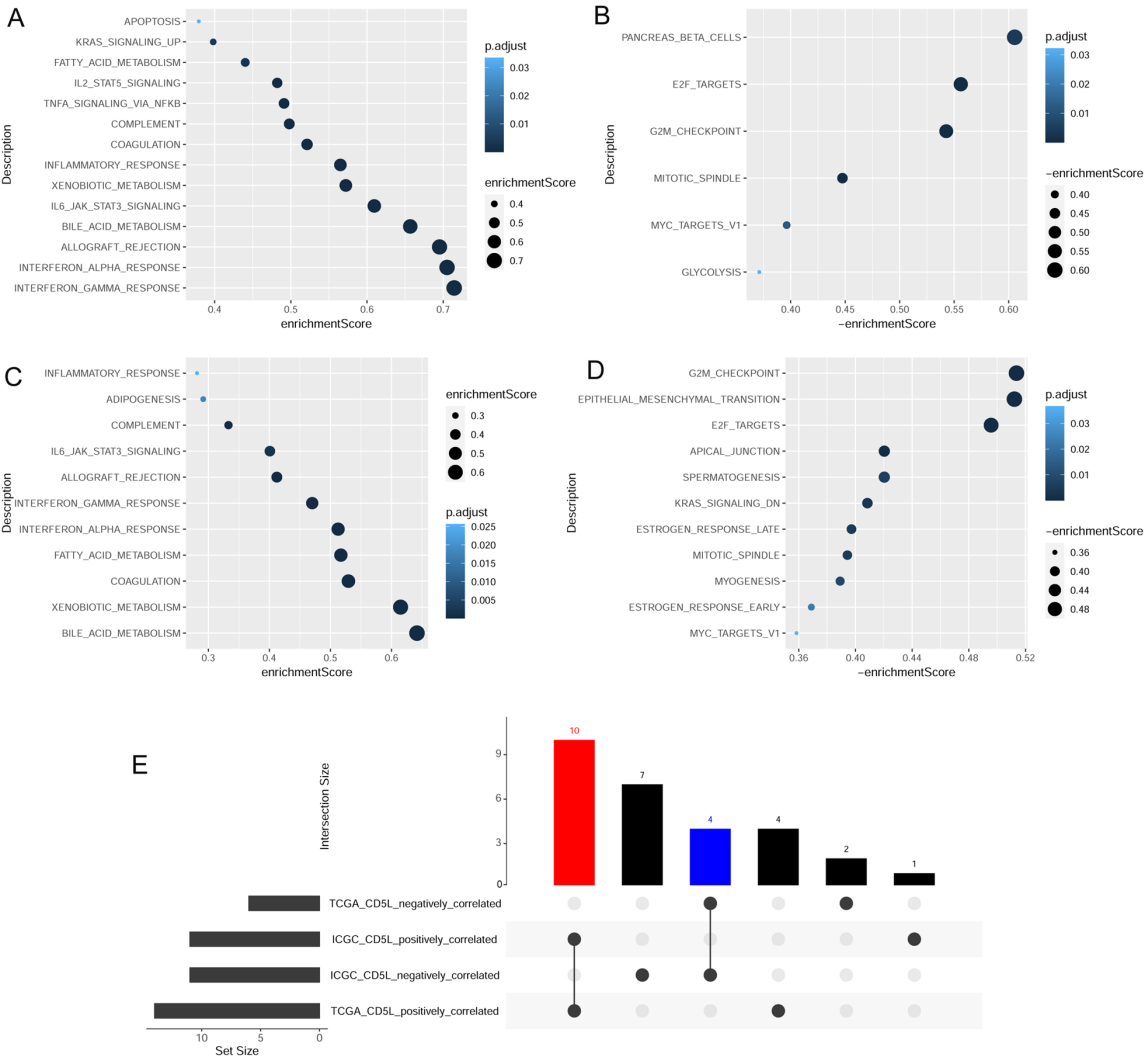
CD5L-correlated pathways in HCC. **A** The pathways positively correlated with CD5L gene expression in HCC samples of TCGA-HCC dataset. **B** The pathways negatively correlated with CD5L gene expression in HCC samples of TCGA-HCC dataset. **C** The pathways positively correlated with CD5L gene expression in HCC samples of ICGC-HCC dataset. **D** The pathways negatively correlated with CD5L gene expression in HCC samples of ICGC-HCC dataset. **E** The common pathways positively (red bar) or negatively (blue bar) correlated with CD5L expression in both of TCGA-HCC and ICGC-HCC datasets. HCC, hepatocellular carcinoma; GSEA, gene set enrichment analysis. GSEA was performed with “GSEABase” package in R and the adjusted *p* < 0.05 was considered significant

With “limma” package in R, the expressional differences of the genes between CD5L^high^ and CD5L^low^ HCC samples in the two datasets were obtained and GSEA was performed in each dataset individually. As shown in Fig. 1, 20 biological processes were shown to be positively (n = 14) or negatively (n = 6) correlated with CD5L expression in HCC in TCGA-HCC dataset (Fig. 1A–B). In ICGC-HCC dataset, 22 biological processes were presented to be positively (n = 11) or negatively (n = 11) correlated with CD5L expression in HCC (Fig. 1C–D). The results in the two datasets were mainly consistent. There were 10 (interferon gamma response, interferon alpha response, allograft rejection, bile acid metabolism, IL-6 JAK stat3 signaling, xenobiotic metabolism, inflammatory response, coagulation, complement, and fatty acid metabolism) and four (MYC targets v1, mitotic spindle, G2M checkpoint, and E2F targets) biological processes (pathways) positively and negatively correlated with CD5L expression in HCC in both datasets (Fig. 1E).

**Fig. 2:**
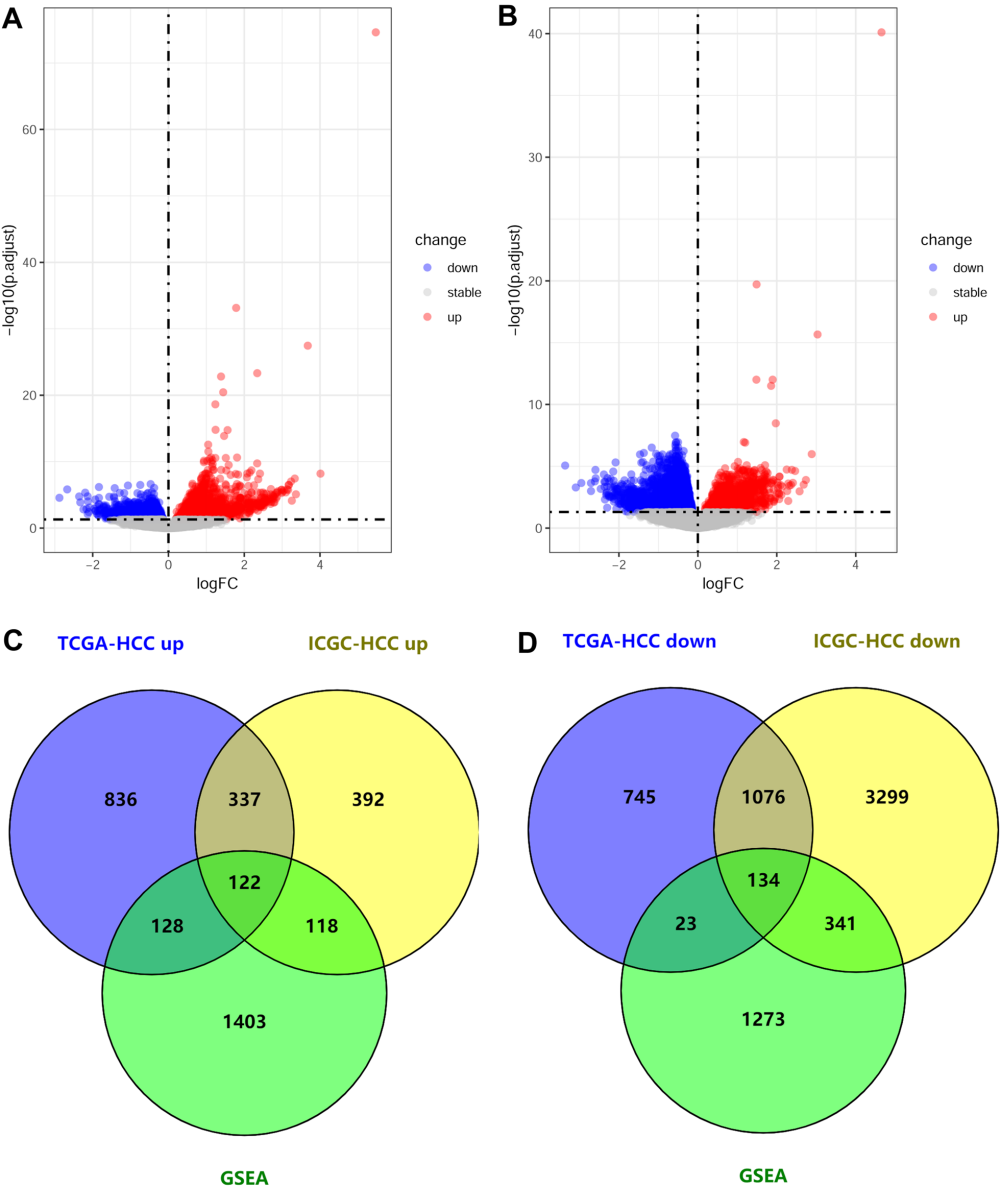
Identification of CD5L-AGs in HCC. **A**–**B** Gene expression comparisons between CD5L^high^ and CD5L^low^ HCC samples in TCGA-HCC dataset and ICGC-HCC dataset, respectively. **C** CD5L-AGs which were both enriched in CD5L-associated pathways and higher expressed in CD5L^high^ HCC samples. **D** CD5L-AGs which were both enriched in CD5L-associated pathways and lower expressed in CD5L^high^ HCC samples. CD5L-AGs, CD5L-associated genes; HCC, hepatocellular carcinoma. “Limma” package in R were used for gene comparisons and adjusted *p* < 0.05 was considered significant

The genes in the 14 CD5L-correlated biological processes were extracted and there were 1771 unique genes. According to the gene expressional differences between CD5L^high^ and CD5L^low^ HCC samples (Fig. 2A–B), 256 of the 1771 genes were higher (n = 122, Fig. 2C) or lower (n = 134, Fig. 2D) expressed in CD5L^high^ HCCs than the CD5L^low^ samples and they were CD5L-AGs.

### Prognostic effects of CD5L-AGs

**Fig. 3:**
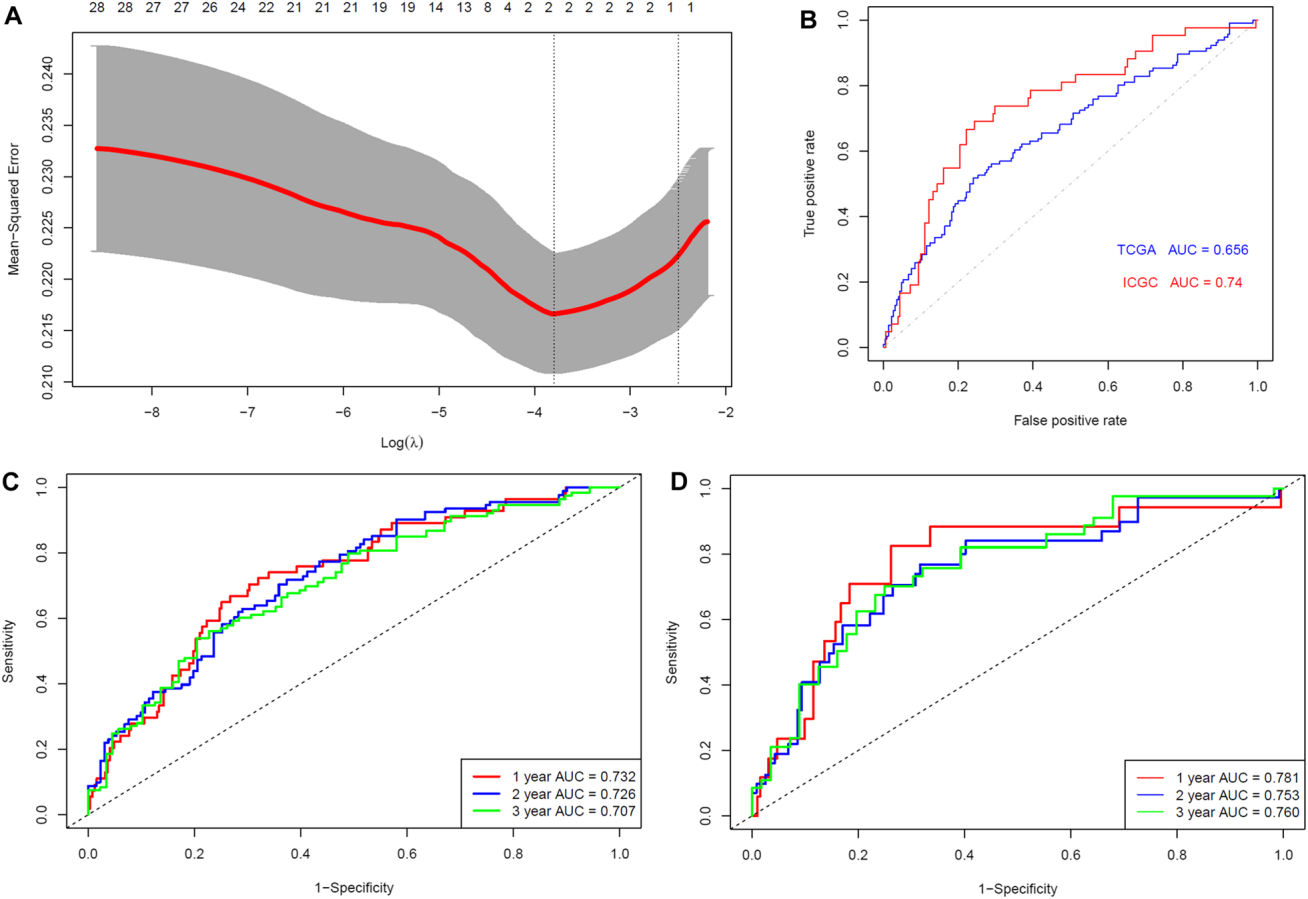
LASSO regression analysis of CD5L-APDGs in HCC. **A** Tuning parameter lambda (λ) selection using tenfold cross validation. **B** The risk model could predicate the survival status with AUCs of 0.656 and 0.740 in TCGA-HCC and ICGC-HCC datasets, respectively. **C**–**D** The efficiency of the risk model in discriminating the 1-year, 2-year and 3-year OS status of TCGA-HCC and ICGC-HCC patients, respectively. CD5L-APDG, CD5L-associated prognostic and diagnostic genes. LASSO, least absolute shrinkage and selection operator. LASSO regression and ROC analyses were performed in R

The gender-age-stage-corrected prognostic effects of the 256 CD5L-AGs were showed in Additional file 1: Table S3 and 84 of them presented favorable or unfavorable prognostic effects in HCC patients in TCGA-HCC and ICGC-HCC datasets and they were CD5L-APGs. According to the gene expression comparisons between HCC and normal liver tissues in TCGA-HCC and ICGC-HCC datasets, most of the CD5L-APGs were shown to be dysregulated in HCC consistently. Among them, 28 differentially expressed genes (Additional file 1: Table S4) meet the criteria of |logFC|> 1 and *p*.adj < 1e-10 and they were called CD5L-APDGs. Through LASSO regression analysis (Fig. 3A), two of the 28 CD5L-APDGs, LCAT and CDC20, were highlighted to be independent prognostic factors. Then, with the coefficients of LCAT and CDC20 deduced from LASSO analysis and their relative expressions, a risk model of HCC OS was constructed as follows:

$$risk score=\,\left(-0.0321\right)*LCAT expression+ 0.0778*CDC20 expression$$

**Fig. 4:**
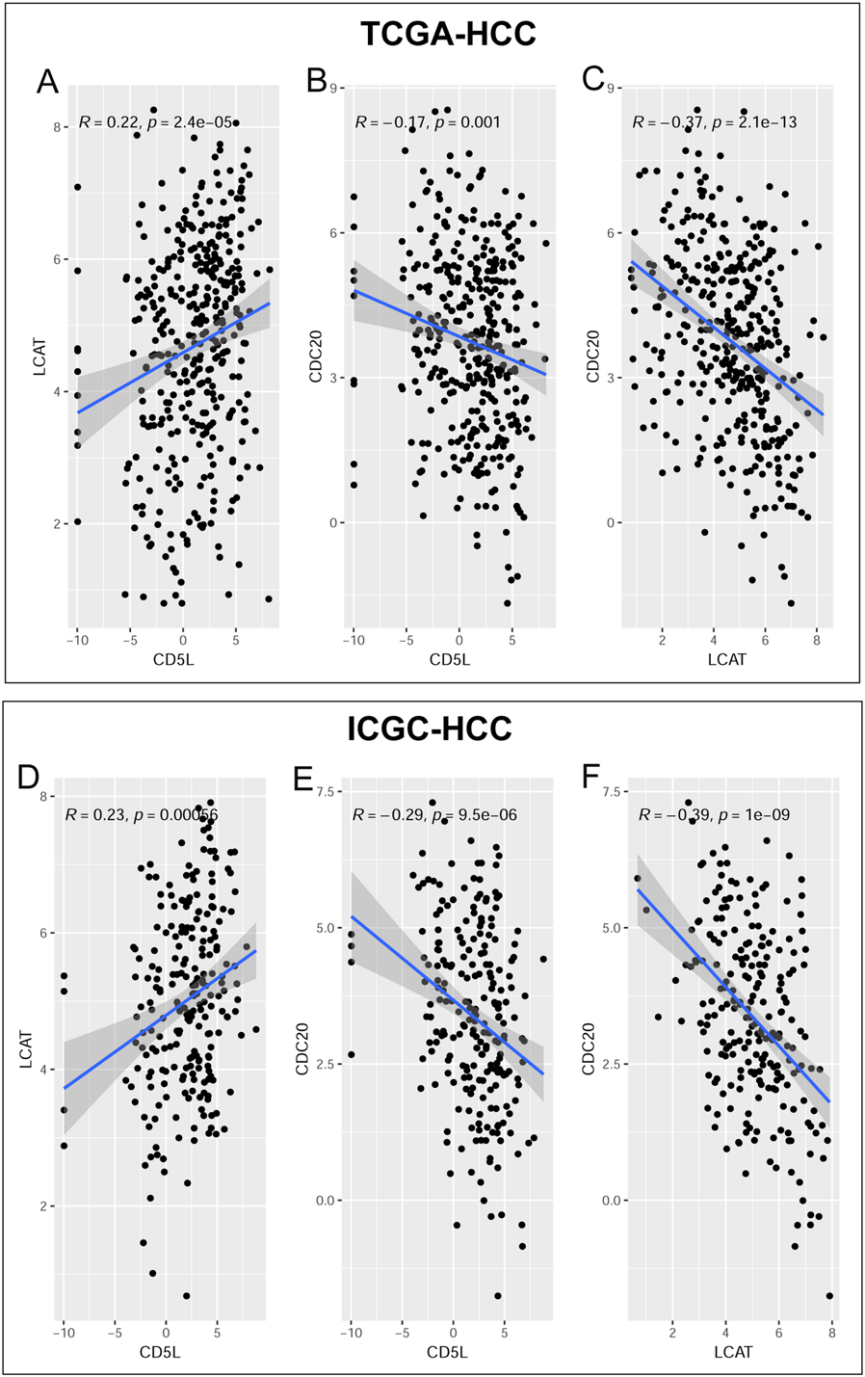
Correlations of LCAT and CDC20 with CD5L in HCC. **A**–**C** Significant correlations between LCAT, CDC20 and CD5L expression in HCC in TCGA-HCC dataset. **D**–**F** Significant correlations between LCAT, CDC20 and CD5L expression in HCC in ICGC-HCC dataset. Spearman correlation analysis was used and *p* < 0.05 was considered significant

According to the ROC analyses, the risk model could discriminate the HCC OS status with an area under the curve (AUC) of 0.656 in TCGA-HCC dataset and the efficiency was confirmed in ICGC-HCC dataset with the AUC of 0.740 (Fig. 3B). In addition, the risk model performed well in predicting the survival status at different time points. As shown in Fig. 3C, in TCGA-HCC dataset, the risk model could discriminate the OS status of HCC patients at 1-year, 2-year and 3-year with an AUC of 0.732, 0.726, 0.707, respectively (Fig. 3C). And the results were similar in ICGC-HCC dataset (Fig. 3D), indicating the effectiveness and stability of the risk model. In a recent study, a five-gene prognostic signature consisted of AURKA, PZP, RACGAP1, ACOT12 and LCAT could discriminate 1-year, 2-year, and 3-year HCC OS status with AUCs of 0.741, 0.724, and 0.718, respectively [37]. Interestingly, here, with less genes, the LCAT-CDC20 risk model presented similar efficiency. The significant positive correlation of LCAT while the negative correlation of CDC20 with CD5L, as well as the negative correlation between LCAT and CDC20, were visualized in Fig. 4. Through Kaplan–Meier survival analysis (Additional file 1: Figure S1**)**, the favorable prognostic effects of LCAT and CD5L while the unfavorable prognostic effects of CDC20 in HCC were visualized.

### Associations of CD5L, LCAT and CDC20 with HCC microenvironment and immunoregulators

Through Wilcoxon tests (Additional file 1: Table S5), immune score, stroma score and microenvironment score as well as 16 kinds of immune/stroma cell infiltrations including macrophages, macrophages M1, macrophages M2, monocytes, and adipocytes were shown to be downregulated in HCC than normal liver controls in both TCGA-HCC and ICGC-HCC datasets. In contrast, 13 kinds of immune/stroma cell infiltration including common lymphoid progenitors (CLP), pro B-cells, Th1 cells, and Th2 cells were decreased in HCC while no significant difference of other kinds of cells were shown in either of the two datasets.

**Fig. 5:**
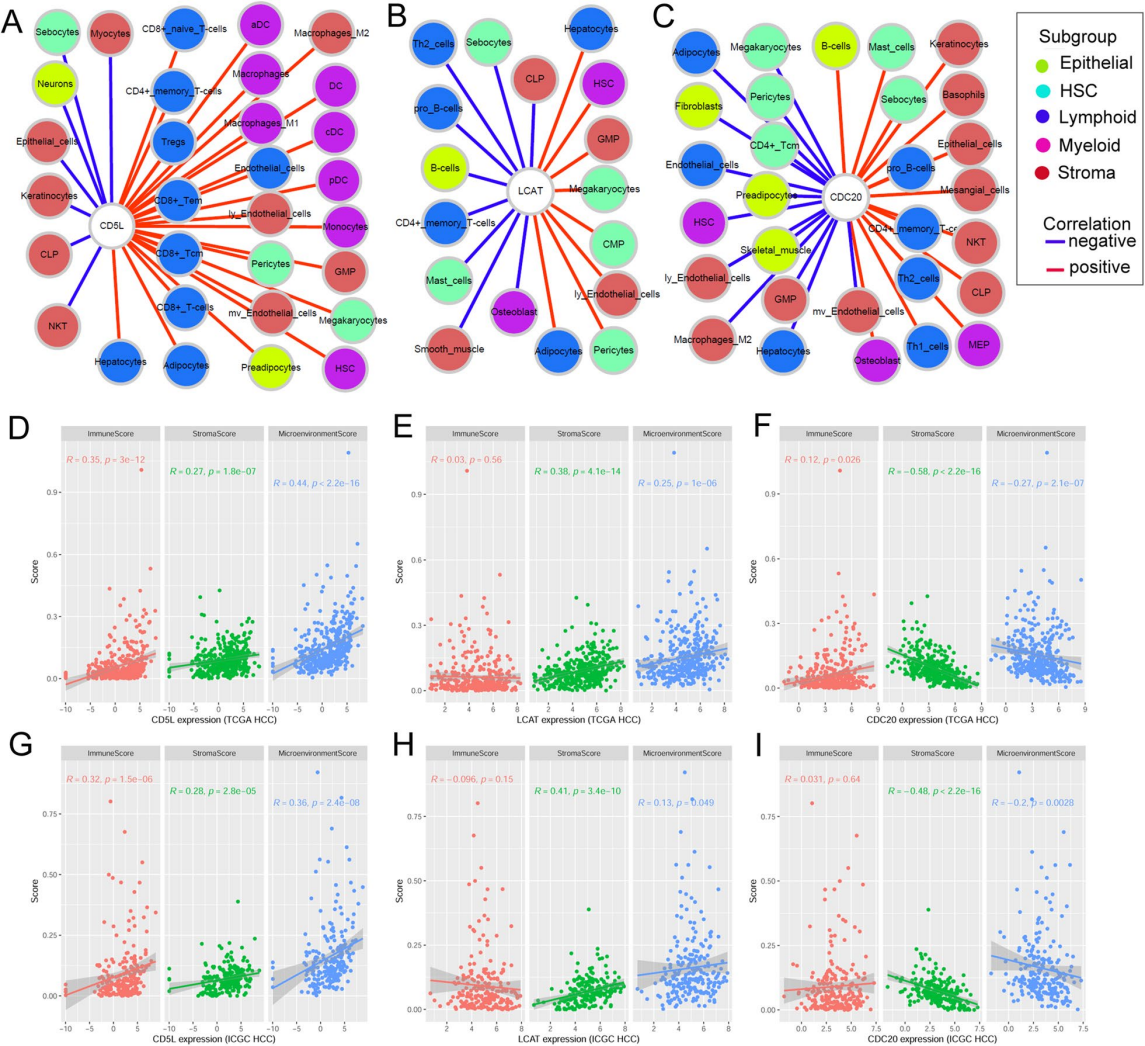
Correlations of CD5L, LCAT, and CDC20 with HCC microenvironment. **A**–**C** Significant correlations of CD5L, LCAT and CDC20 with immune and stroma cell infiltrations in HCC. **D**–**F** Correlations of CD5L, LCAT and CDC20 with immune score, stroma score and microenvironment score in HCC in TCGA-HCC dataset. **G**–**I** Correlations of CD5L, LCAT and CDC20 with immune score, stroma score and microenvironment score in HCC in ICGC-HCC dataset. Spearman correlation analysis was used and *p* < 0.05 was statistically significant

Through correlation analyses, most (45/64) of the immune and stroma cells were shown to have significant positive or negative correlations with at least one of the three genes in HCC in both TCGA-HCC and ICGC-HCC datasets (Fig. 5A–C, Additional file 1: Table S6). As shown in Fig. 5D and G, CD5L was shown to be positively correlated with immune score, stroma score and microenvironment score. Although LCAT (Fig. 5E and H) and CDC20 (Fig. 5F, I) presented significant positive/negative correlations with microenvironment score, it was stroma score (*p* < 0.01) but not immune score (*p* > 0.05) which indicated significant correlations with them.

**Fig. 6:**
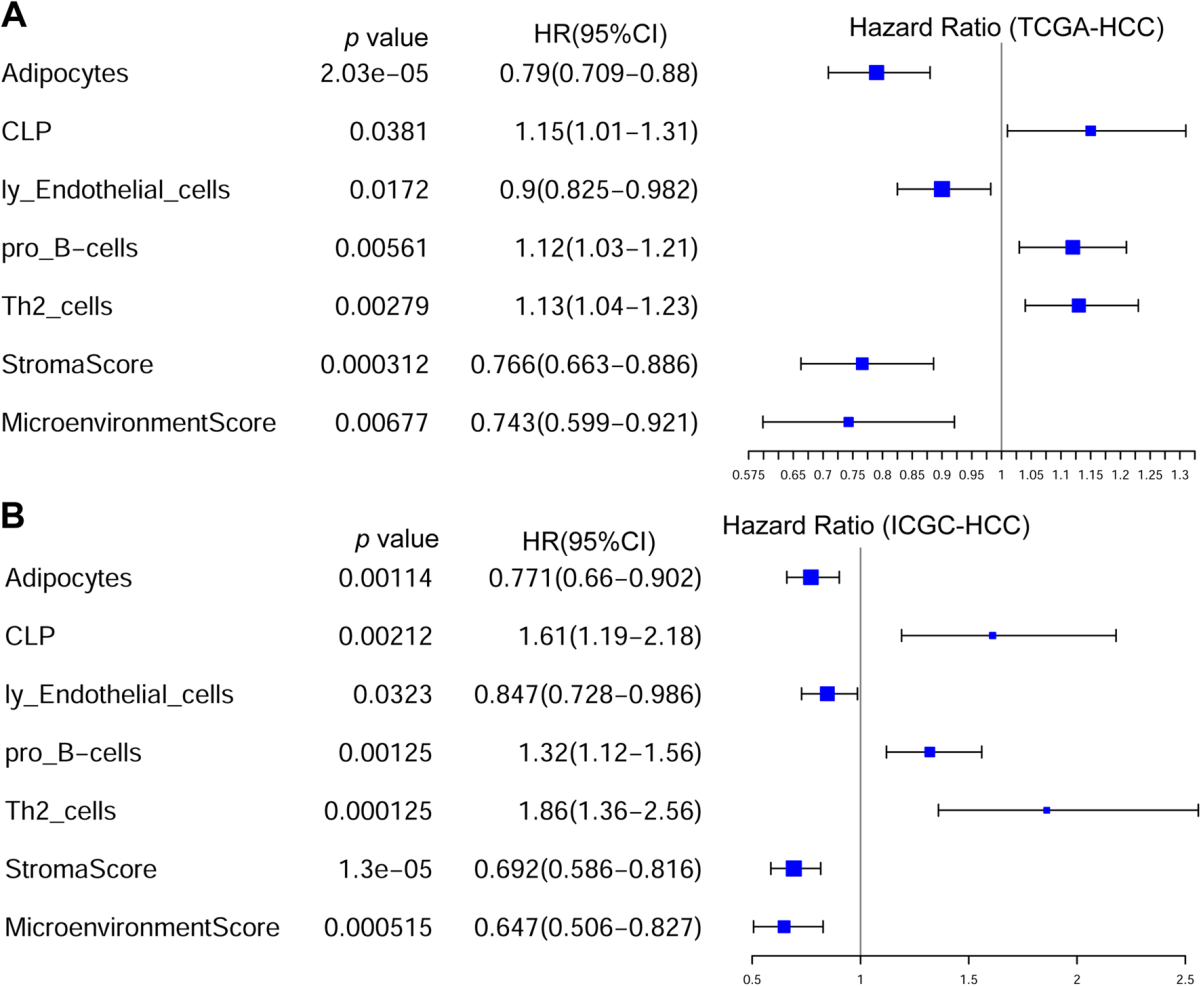
Prognostic effects of microenvironment on HCC OS. **A** Prognostic effects of immune and stroma cells on HCC OS in TCGA-HCC dataset. **B** Prognostic effects of immune and stroma cells on HCC OS in ICGC-HCC dataset. OS, overall survival. Multi-variable Cox regression analysis was performed and the age-gender-stage-corrected prognostic effects were evaluated. *P* < 0.05 was considered statistically significant

With multivariable Cox regression analyses (Fig. 6, Additional file 1: Table S7), among the immune and stroma cells correlated with CD5L, LCAT, or CDC20, the abundances of adipocytes, CLP, lymphatic endothelial cells, pro-B cells, and Th2 cells were shown to be prognostic factors independent of gender, age and stage. For the scores, only the stroma score and the microenvironment score (Fig. 6) were indicated to be favorable prognostic factors for HCC OS while no significance of immune score (Additional file 1: Table S7) was shown.

**Fig. 7:**
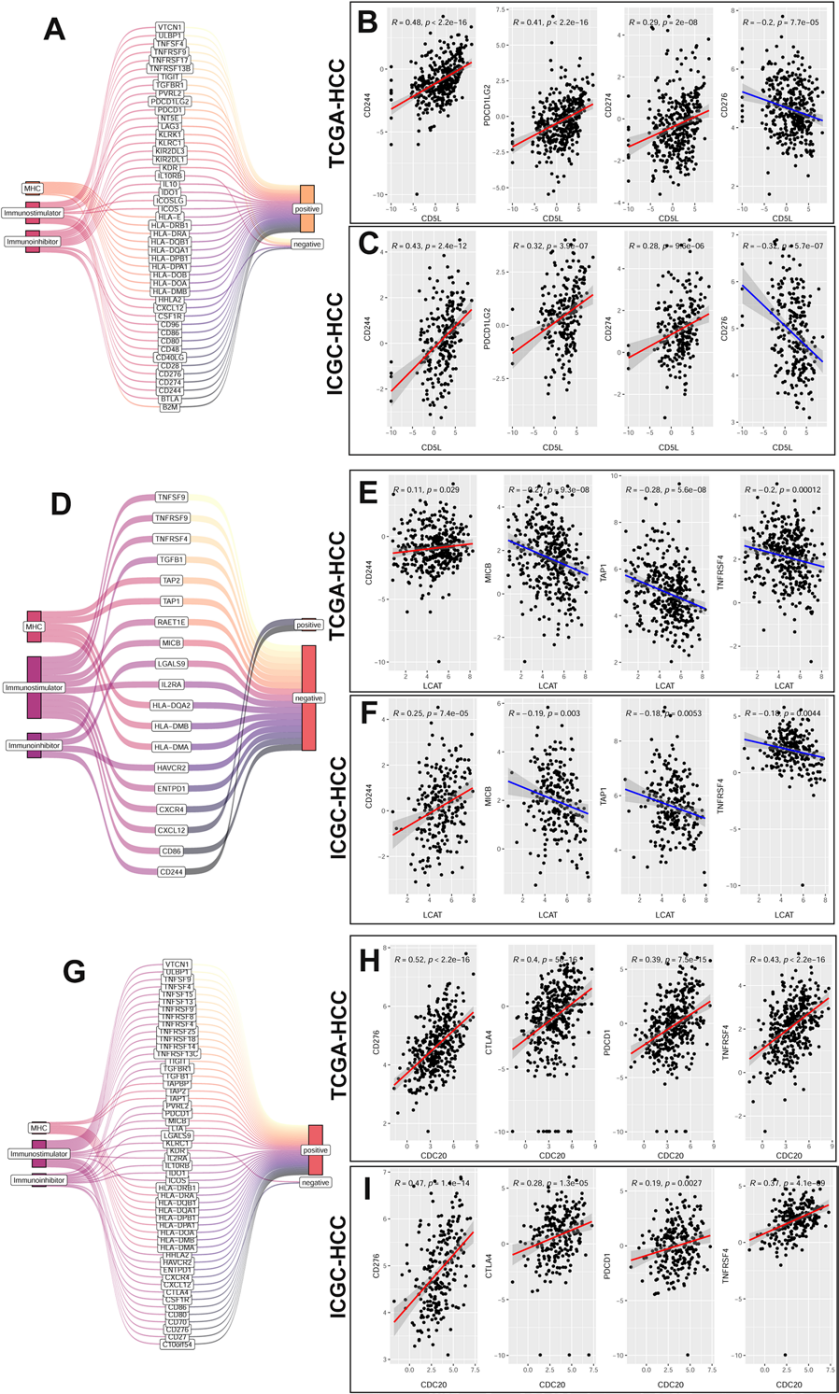
The correlations of CD5L, LCAT and CDC20 with immunoregulators. **A** The significant correlations of CD5L with 18 immunoinhibitors, 18 immunostimulators and 11 MHC-related genes in HCC. **B**–**C** The significant positive correlations of CD5L with CD244, PDCD1LG2, CD274 (Programmed death ligand 1, PD-L1) while negative correlation with CD276 (B7-H3) in TCGA-HCC and ICGC-HCC, respectively. **D** The significant correlations of LCAT with four immunoinhibitors, ten immunostimulators and five MHC-related genes in HCC. **E**–**F** The positive correlation of LCAT with CD244 while negative correlations with MICB, TAP1, and TNFRSF4 in TCGA-HCC and ICGC-HCC, respectively. **G** The significant correlations of CDC20 with 13 immunoinhibitors, 27 immunostimulators and 12 MHC-related genes in HCC. **H**–**I** The positive correlations of CDC20 with CD276, CTLA4, PDCD1, and TNFRSF4 TCGA-HCC and ICGC-HCC, respectively. PDCD1LG2, programmed cell death 1 ligand 2; MICB, MHC class I polypeptide-related sequence B; TAP1, transporter 1; TNFRSF4, TNF receptor superfamily member 4; CTLA4, cytotoxic T-lymphocyte associated protein 4. Spearman correlation analysis was used and *p* < 0.05 was considered significant

As shown in Fig. 7, the significant correlations of CD5L, LCAT, and CDC20 with immunoregulators in HCC samples in both TCGA-HCC and ICGC-HCC datasets were shown. There were 18 immunoinhibitors, 18 immunostimulators and 11 MHC-related genes positively (n = 39) or negatively (n = 8) correlated with CD5L expression in both TCGA-HCC and ICGC-HCC datasets (Fig. 7A). Among them, the significant positive correlations of CD5L with CD244, programmed cell death 1 ligand 2 (PDCD1LG2), CD274 (programmed death ligand 1, PD-L1) while negative correlation with CD276 (B7-H3) were obvious (Fig. 7B–C). As shown in Fig. 7D, there were four immunoinhibitors, ten immunostimulators and five MHC-related genes negatively (n = 17) or positively (n = 2, CD244 and CXCL12) correlated with LCAT expression. The positive correlation of LCAT with CD244 while its negative correlations with MHC class I polypeptide-related sequence B (MICB), Transporter 1 (TAP1, an ATP binding cassette subfamily B member) and TNF receptor superfamily member 4 (TNFRSF4) were shown in Fig. 7E–F. For CDC20, it was shown to be positively (n = 20) or negatively (n = 2, KDR and CXCL12) correlated with 13 immunoinhibitors, 27 immunostimulators and 12 MHC-related genes in HCC (Fig. 7G). The positive correlations of CDC20 with CD276, cytotoxic T-lymphocyte associated protein 4 (CTLA4), PDCD1, and TNFRSF4 were visualized in Fig. 7H–I.

CD5L, LCAT and CDC20 also presented significant correlations with NK-κB associated genes in HCC samples (Additional file 1: Figure S2). As shown in Additional file 1: Figure S2A–B, there was a significant negative correlation between CD5L expression and RELB expression in both TCGA-HCC (*r* = −0.16, *p* < 0.01) and ICGC-HCC (*r* = −0.13, *p* < 0.05) samples. Similarly, consistent negative correlations of LCAT expression with NFKB1 (TCGA-HCC: *r* = −0.17, *p* < 0.01; ICGC-HCC: *r* = −0.16, *p* < 0.05) and RELB (TCGA-HCC: *r* = −0.3, *p* < 0.01; ICGC-HCC: *r* = −0.17, *p* < 0.05) expressions were shown. In contrast, CDC20 presented consistent positive correlations of with NKFB1 (TCGA-HCC: *r* = 0.17, *p* < 0.01; ICGC-HCC: *r* = 0.14, *p* < 0.05), NFKB2 (TCGA-HCC: *r* = 0.4, *p* < 0.01; ICGC-HCC: *r* = 0.22, *p* < 0.01), RELA (TCGA-HCC: *r* = 0.35, *p* < 0.01; ICGC-HCC: *r* = 0.31, *p* < 0.01), and RELB (TCGA-HCC: *r* = 0.43, *p* < 0.01; ICGC-HCC: *r* = 0.35, *p* < 0.01) expressions in HCC samples. For the associations of CD5L, LCAT and CDC20 with chemokines and chemokine receptors in HCC, similar results were shown in TCGA-HCC (Additional file 1: Figure S3A) and ICGC-HCC (Additional file 1: Figure S3B) datasets. CD5L presented consistent positive correlations with 18 chemokines and 12 chemokine receptors while negative correlations with four chemokines in the two HCC datasets. For LCAT, its consistent positive correlations with five chemokines while negative correlations with five other chemokines and three chemokine receptors were shown. With regard to CDC20, its consistent positive correlations with 17 chemokines and eight chemokine receptors while negative correlation with four chemokines in HCC samples were obvious. These results also indicated the immunoregulatory potential of the three genes in HCC.

### The associations of CD5L, LCAT and CDC20 with the sensitivity of HCC cell lines to anti-cancer drugs

There were 175 anti-cancer drugs for HCC cell lines and there were 11 HCC cell lines which had the gene expression data and the pharmacologic data of anti-cancer drugs. As there was only one kind of HCC cell line which had the pharmacologic data about the drug OSI-027, 174 of the anti-drugs were included for further analysis and their LN_IC50s were investigated. The correlations of the three genes expression with the 174 anti-cancer drugs in HCC cell lines were shown in (Additional file 2). CD5L expression was significantly negatively correlated with the LN_IC50s of Mitoxantrone, OTX015, and I-BRD9 while positively correlated that of Fulvestrant (Table 2)**,** indicating its positive/negative correlations with the sensitivity of HCC cell lines to the four drugs. LCAT expression was significantly negatively correlated with the LN_IC50s of seven anti-cancer drugs (IGF1R_3801, WZ4003, Niraparib, NVP-ADW742, AZD5582, and Alpelisib) while positively correlated LN_IC50s of four anti-cancer drugs (Acetalax, P22077, AZD4547, Sorafenib, and Dabrafenib) (Table 2)**,** indicating its positive/negative correlations with the sensitivity of HCC cell lines to the eleven drugs. For CDC20, its negative correlations with the LN_IC50s of 18 anti-cancer drugs were shown (Table 2), indicating its positive correlation with the sensitivities of HCC to the 18 anti-cancer drugs.

**Table 2:** The correlations of CD5L, LCAT and CDC20 with anti-cancer drug sensitivities in HCC cell lines

	Drugs	Putative target	Pathway name	R	P value
CD5L	Mitoxantrone		DNA replication	−0.811	0.002^**^
	OTX015	BRD2, BRD3, BRD4	Chromatin other	−0.642	0.033^*^
	I-BRD9	BRD9	Chromatin other	−0.621	0.041^*^
	Fulvestrant	ESR	Hormone-related	0.642	0.033^*^
LCAT	IGF1R_3801	IGFR1	IGF1R signaling	−0.755	0.010^*^
	WZ4003	NUAK1, NUAK2	Other, kinases	−0.691	0.023^*^
	Niraparib	PARP1, PARP2	Genome integrity	−0.682	0.025^*^
	NVP-ADW742	IGF1R	IGF1R signaling	−0.645	0.037^*^
	AZD5582	XIAP, cIAP	Apoptosis regulation	−0.627	0.044^*^
	Alpelisib	PI3Kalpha	PI3K/MTOR signaling	−0.618	0.048^*^
	Sorafenib	PDGFR, KIT, VEGFR, RAF	Other, kinases	0.636	0.040^*^
	Dabrafenib	BRAF	ERK MAPK signaling	0.736	0.013^*^
	AZD4547	FGFR1, FGFR2, FGFR3	RTK signaling	0.755	0.010^*^
	P22077	USP7, USP47	Protein stability and degradation	0.800	0.005^**^
	Acetalax		Unclassified	0.809	0.004^**^
CDC20	Picolinici-acid	Inflammatory related	Other	−0.873	0.001^**^
	Vincristine		Mitosis	−0.818	0.004^**^
	Niraparib	PARP1, PARP2	Genome integrity	−0.745	0.012^*^
	Palbociclib	CDK4, CDK6	Cell cycle	−0.727	0.015^*^
	Carmustine		DNA replication	−0.727	0.015^*^
	PRIMA-1MET	TP53 activation	p53 pathway	−0.718	0.017^*^
	Fludarabine		Unclassified	−0.709	0.019^*^
	CZC24832	PI3Kgamma	PI3K/MTOR signaling	−0.700	0.021^*^
	AZD5363	AKT1, AKT2, AKT3, ROCK2	Other, kinases	−0.682	0.025^*^
	Docetaxel	Microtubule stabiliser	Mitosis	−0.682	0.025^*^
	CDK9_5038	CDK9	Cell cycle	−0.673	0.028^*^
	Mitoxantrone		DNA replication	−0.673	0.028^*^
	Vinblastine	Microtubule destabiliser	Mitosis	−0.664	0.031^*^
	Vinorelbine	Microtubule destabiliser	Mitosis	−0.645	0.037^*^
	JAK_8517	JAK1, JAK2	Other, kinases	−0.636	0.040^*^
	Dinaciclib	CDK1, CDK2, CDK5, CDK9	Cell cycle	−0.627	0.044^*^
	Podophyllotoxin bromide		Unclassified	−0.627	0.044^*^
	Dactinomycin		Other	−0.627	0.044^*^

^*^p < 0.05; ^**^p < 0.01. The correlations of the gene expressions with log-transformed half maximal inhibitory concentrations (LN_IC50s) of the drugs in the HCC cell lines were estimated. Spearman correlation analysis was used and *p* < 0.05 was considered significant

### Validation of the dysregulation of CD5L, LCAT and CDC20 in HCC at protein level

**Fig. 8:**
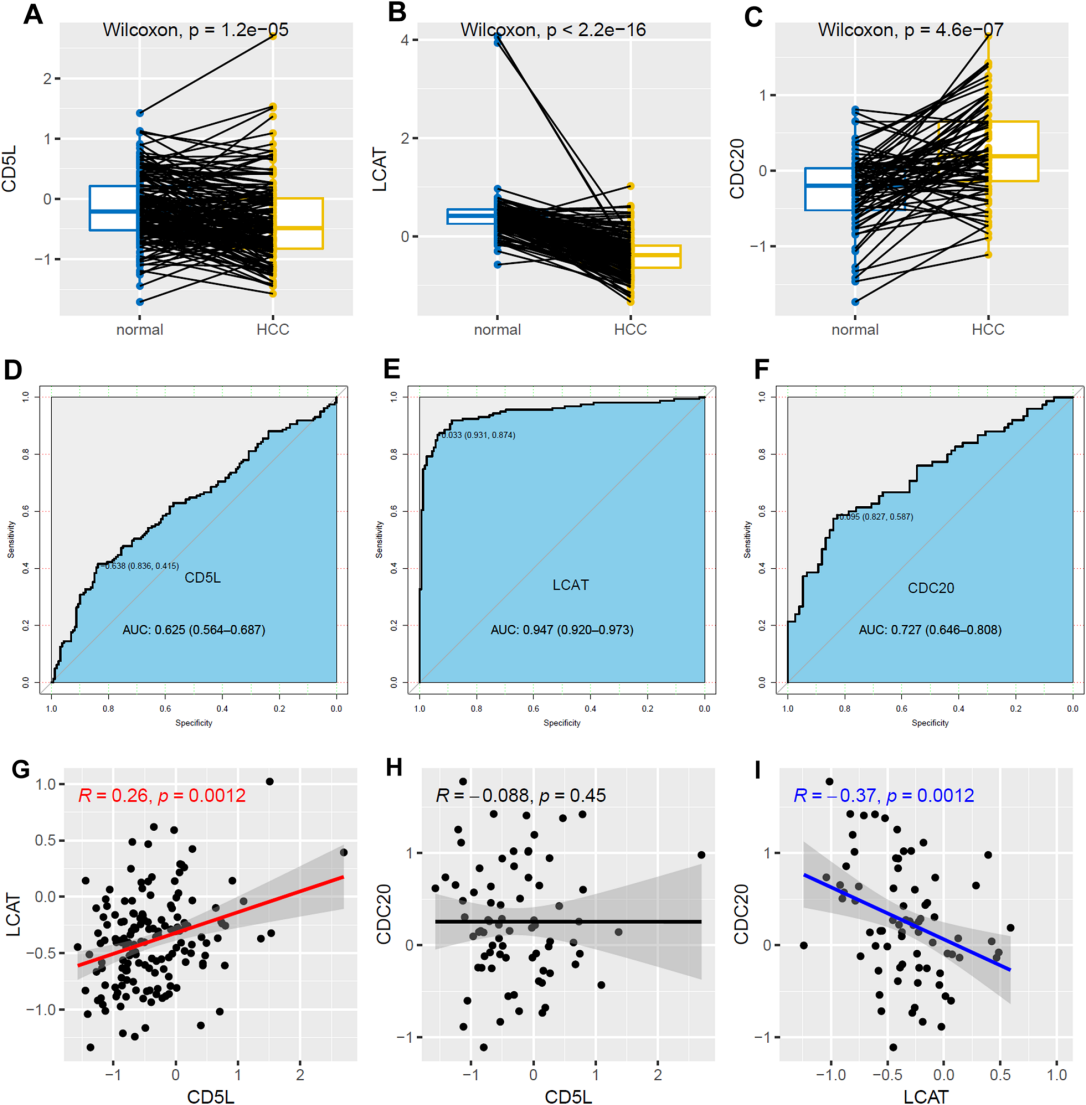
The dysregulations, diagnostic power and correlations of CD5L, LCAT and CDC20 in HCC. **A** Lower CD5L in HCC tumors than their paired controls at protein level. **B** Lower LCAT in HCC tumors than their paired controls at protein level. **C** Higher CD5L expression in HCC tumors than their paired controls at protein level. **D**–**F** ROC analyses of CD5L, LCAT and CDC20 in discriminating HCC tumors and normal liver controls. **G**–**I** Spearman correlation analyses of CD5L, LCAT and CDC20 expressions in HCC at protein level. ROC, receiver operating characteristic. Two-sided paired Wilcoxon test, ROC analysis and Spearman analysis were used and *p* < 0.05 was considered significant

At protein level, as shown in Fig. 8, CD5L (Fig. 8A) and LCAT (Fig. 8B) were shown to be lower while CDC20 (Fig. 8C) higher expressed in HCC tissues than their paired normal liver tissues, consistent with their dysregulations at mRNA level. Considering the significant difference between the T stage proportions between CPTAC and TCGA HCC samples (Additional file 1: Figure S4A), the expressional differences between HCC samples of different stages and the normal samples were also compared (Additional file 1: Figure S5). Comparing with normal samples, CD5L (Additional file 1: Figure S5A) and LCAT (Additional file 1: Figure S5B) presented consistent lower expressions while CDC20 (Additional file 1: Figure S5C) presented consistent up-regulation in both T1/T2 tumors and T3/T4 tumors. Through ROC analysis (Fig. 8D-F), the diagnostic potential of the three proteins in HCC were shown, with AUCs of 0.625, 0.947 and 0.727 for CD5L, LCAT and CDC20, respectively. With regard to their correlations, LCAT was shown to be positively with CD5L expression (Fig. 8G) while negatively correlated with CDC20 expression (Fig. 8I) in the HCC tumors. However, in contrast to the negative correlation between CD5L and CDC20 at mRNA level, no significant correlation between them was found at protein level (Fig. 8H).

**Fig. 9:**
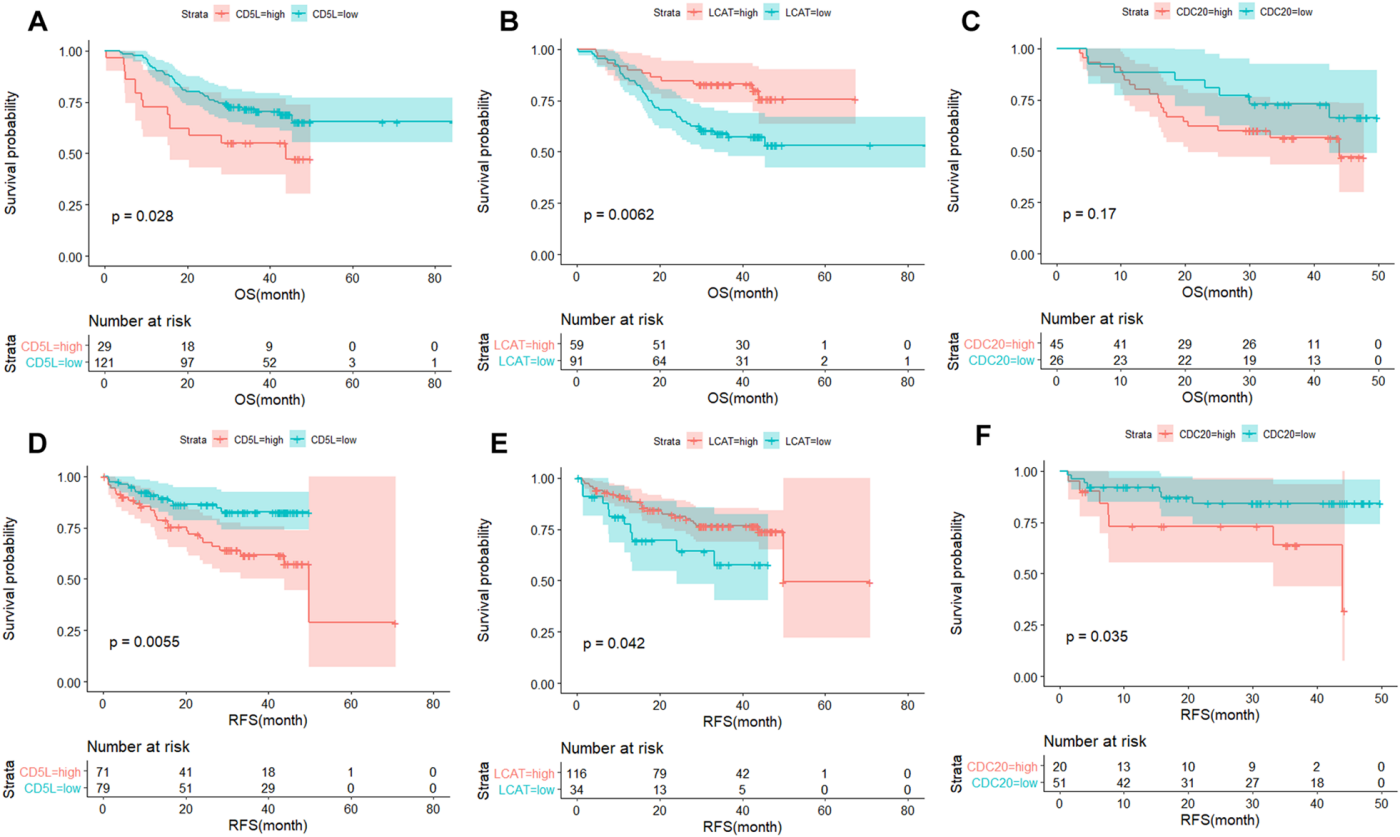
Kaplan–Meier survival analyses of CD5L, LCAT and CDC20 in HCC at protein level. **A**–**C** Prognostic effects of CD5L, LCAT and CDC20 on HCC OS in HCC at protein level. **D**–**F** Prognostic effects of CD5L, LCAT and CDC20 on HCC RFS in HCC at protein level. OS, overall survival; RFS, relapse-free survival. Kaplan–Meier survival analysis with log-rank test was used and *p* < 0.05 was considered significant

The prognostic effects of the three proteins were also evaluated in the HCC patients. Surprisingly, in contrast to its favorable prognostic effects on HCC OS at mRNA level, CD5L protein showed significant unfavorable prognostic effects on HCC OS (*p* = 0.028, Fig. 9A) and replase-free survival (RFS) (*p* = 0.0055, Fig. 9D). Consistent with its favorable prognostic effects on HCC OS at mRNA level, LCAT protein presented favorable prognostic effects on HCC OS (*p* = 0.0062, Fig. 9B) and RFS (*p* = 0.042, Fig. 9E). For CDC20 protein, its unfavorable prognostic effects on HCC RFS (*p* = 0.035, Fig. 9F) were presented. Although no statistically significant prognostic effects of CDC20 was shown on HCC OS at protein level, the potential adverse effect of high-CDC20 expression on the long-term survival (survival time  > 15 months) of patients was indicated from the OS survival curves of the two groups (Fig. 9C).

**Fig. 10:**
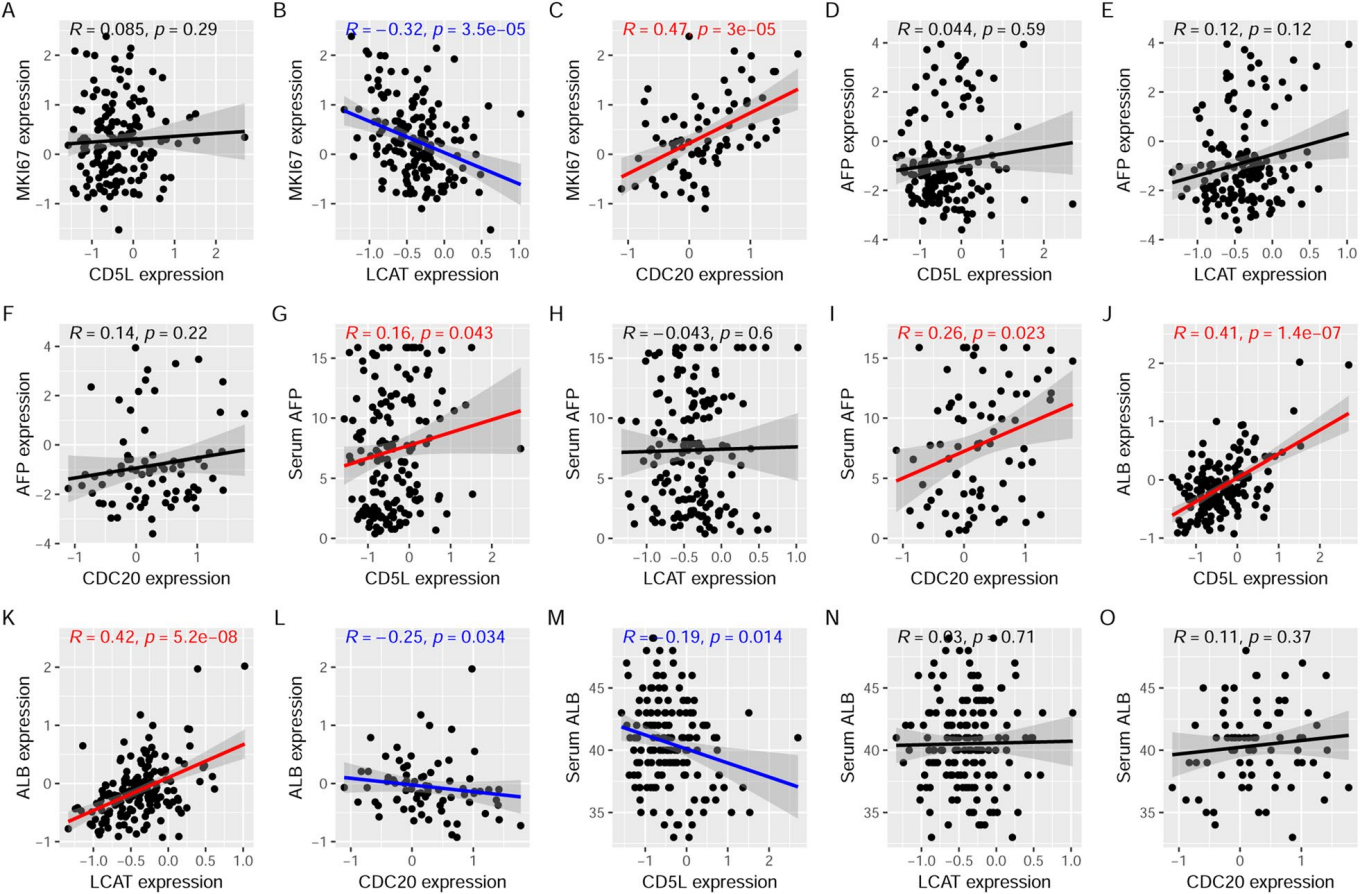
Correlations of CD5L, LCAT, and CDC20 with MKI67, APF, and ALB in HCC. **A**–**C** The correlations of CD5L, LCAT and CDC20 with MKI67 expression in HCC. **D**–**F** The correlations of CD5L, LCAT and CDC20 expressions with AFP expression (tissue AFP) in HCC. **G**–**I** The correlations of CD5L, LCAT and CDC20 expressions with serum AFP (ng/ml) in HCC. **J**–**L** The correlations of CD5L, LCAT and CDC20 expressions with ALB expression (tissue ALB) in HCC. **M**–**O** The correlations of CD5L, LCAT and CDC20 expressions with serum ALB (g/l) in HCC. For **G**–**I**, the serum AFP level was log_2_(x) transformed. Spearman correlation analysis was used and *p* < 0.05 was considered significant

As shown in Fig. 10, LCAT (*r* = −0.32, *p* < 0.01, Fig. 10B) and CDC20 (*r* = 0.47, *p* < 0.01, Fig. 10C) expressions presented significant correlations with MKI67 expression while there was no significant correlation between CD5L expression and MKI67 expression (*r* = 0.085, *p* = 0.29, Fig. 10A). There was a significant positive correlation between serum AFP and AFP expression (tissue AFP) in HCC patients while no significant correlation between serum ALB and ALB expression (tissue ALB) was shown (Additional file 1: Figure S6). Interestingly, although there was no significant correlation between tissue AFP and the three proteins (Fig. 10D-F, p > 0.05), CD5L and CDC20 presented positive correlations with serum AFP (*p* < 0.05, Figs. 10G and I). However, no significant correlation was shown between LCAT expression and serum AFP (*p* > 0.05, Fig. 10H). As shown in Fig. 10J-L, CD5L (*r* = 0.41, *p* < 0.01) and LCAT (*r* = 0.42, *p* < 0.01) expressions presented significant positive correlations while CDC20 expression was negatively correlated (*r* = −0.25, *p* < 0.05) with tissue ALB in HCC patients. However, with regard to their associations with serum ALB, a significant negative correlation (*r* = −0.19, *p* < 0.05, Fig. 10M) of CD5L expression with serum ALB was shown while there was no significant correlation of LCAT expression and CDC20 expression with serum ALB (*p* > 0.05, Fig. 10N–O). The opposite correlations of CD5L expression with tissue ALB and serum ALB might be associated with the secretion of ALB into the blood. For LCAT and CDC20, the different correlations indicated that they are more closely associated with tissue ALB than with serum ALB.

According to the subcellular information of the proteins in HPA, no CD5L was shown inner the cells (Additional file 1: Figure S7A). Interestingly, as a secretory protein, LCAT was also detected in nucleoplasm (Additional file 1: Figure S7B). For CDC20, its detection was shown in the nucleoplasm and cytosol (Additional file 1: Figure S7C). The location of CD5L was confirmed in liver cells and HCC tumor cells in HPA database. As shown in Additional file 1: Figure S8A-D), although it was not found in the liver cells and HCC cells, CD5L was stained positive in the stroma of all the liver samples (n = 3) while a proportion (3/7) of HCC samples, consistent with its secretory characteristics. Combining with the lower expression of CD5L in HCC tissues than normal liver tissues in CPTAC-HCC dataset, the lower expression of CD5L in HCC stroma was deduced and the important associations of CD5L with HCC microenvironment were indicated. For CDC20, its positive staining in HCC while negative expression in liver was shown (Additional file 1: Figure S8E–H). Although there was no IHC data in HPA, its lower expression in HCC tissues than liver samples were reported in a previous study [38].

### Detection of serum CD5L and CDC20 in HCC and normal controls

**Fig. 11:**
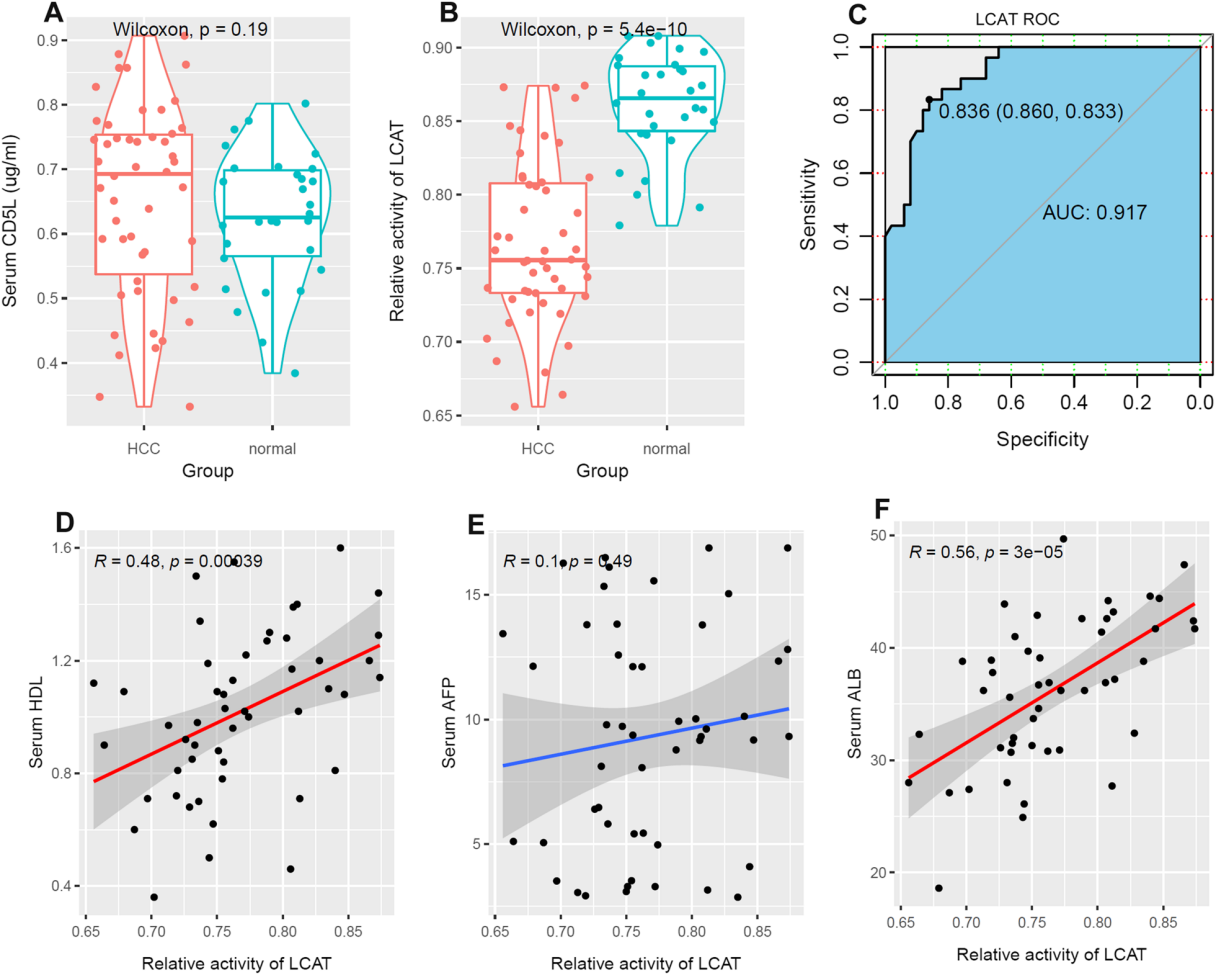
Comparisons of serum CD5L concentration and LCAT activity between HCC patients and normal controls. **A** There was no significant difference of serum CD5L concentration between HCC patients and normal controls. **B** The relative activity of LCAT was lower in HCC serum than normal serum. **C** ROC analysis of serum LCAT in discriminating HCC and normal controls. **D** Significant positive correlation between serum LCAT activity and HDL concentration in HCC. **E** No significant correlation between serum LCAT activity and AFP concentration in HCC. **F** Significant positive correlation between serum LCAT activity and ALB concentration in HCC. Wilcoxon test, ROC analysis, and Spearman correlation analysis were used and *p* < 0.05 was considered significant

The T stage proportions of HCC samples in Henan dataset were similar to the CPTAC dataset (Additional file 1: Figure S4B). According to the ELISA detection (Additional file 3), the CD5L concentration was ranging from 0.332 µg/ml to 0.907 µg/ml (median: 0.693µg/ml) in HCC serum and ranging from 0.384µg/ml to 0.802µg/ml (median: 0.626µg/ml) in normal serum. However, as shown in Fig. 11A, there was no significant difference of CD5L concentrations between HCC samples and normal controls (*p* > 0.05). In contrast, comparing with normal controls, lower activity of LCAT was shown in HCC sera (*p* < 0.01, Fig. 11B), consistent with its lower expression in HCC tissues. Through ROC analysis, the relative activity of LCAT could discriminate HCC samples from normal controls with an AUC of 0.917 (Fig. 11C), indicating its diagnostic potential. In addition, serum LCAT activity was positively correlated with HDL (*R* = 0.48, *p* < 0.01, Fig. 11D) and ALB (*R* = 0.56, *p* < 0.01, Fig. 11F) concentrations in HCC while no significant correlation between LCAT activity and serum AFP (*p* > 0.05, Fig. 11E) was shown.

### The protein-chemical interaction network of CD5L, CAT and CDC20

**Fig. 12:**
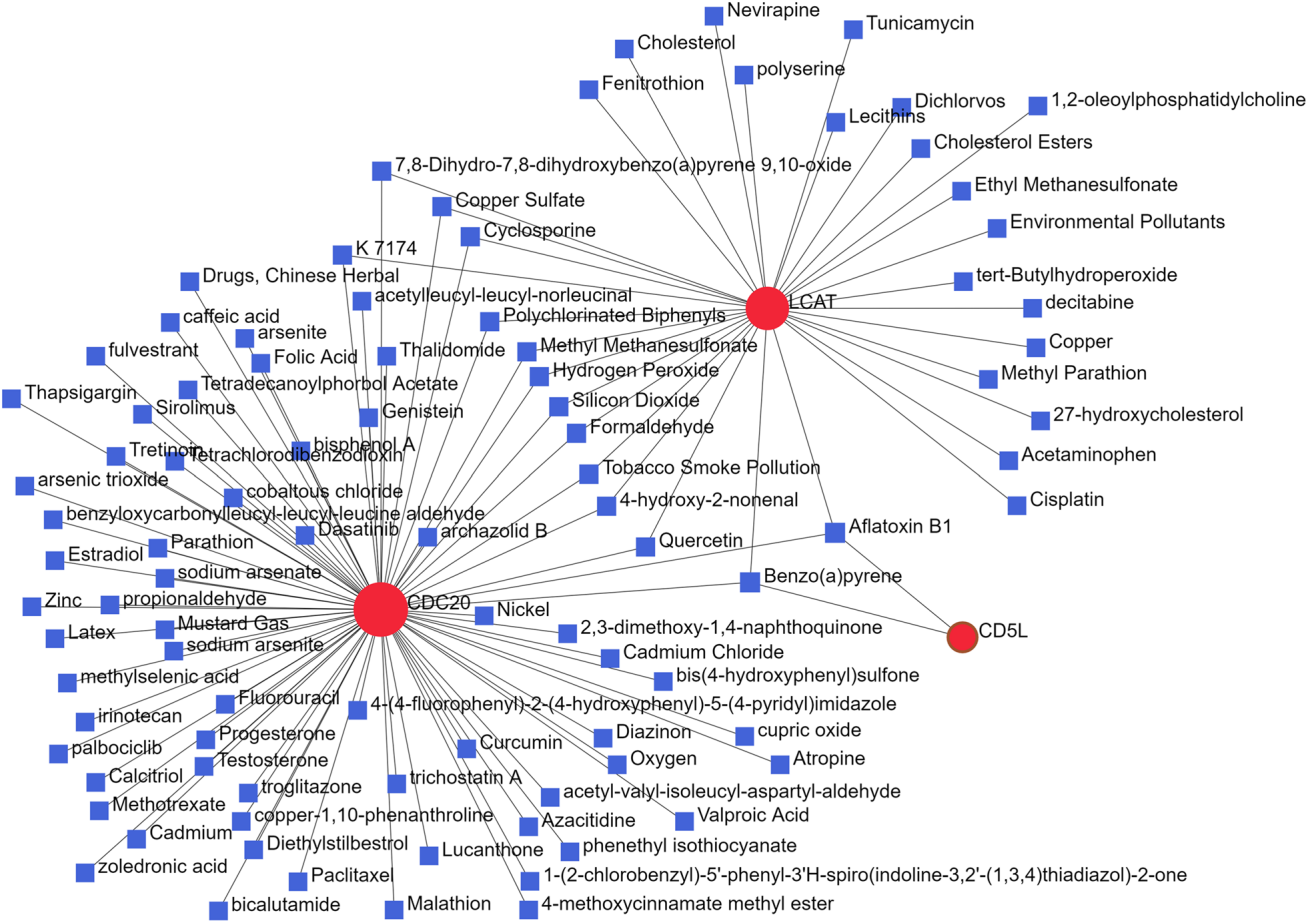
Protein-chemical interactions of CD5L, LCAT and CDC20. The red and blue nodes indicated the proteins and the chemicals, respectively. The edges represented the protein-chemical interactions

As shown in Fig. 12, for CD5L, CAT and CDC20, there were 93 chemicals and 109 protein-chemical interactions. Interestingly, two HCC-associated chemicals, aflatoxin B1 [39] and benzo(a)pyrene [40], were found to be associated with all of the three proteins. These interactions might provide new clues for HCC study.

## Discussion

The dysregulation of lipid metabolism and its associations with immune response were reported in many cancers [41]. In HCC, the close relation between aberrant lipid metabolism and the immune microenvironment was also reported [13]. Considering the regulatory roles of CD5L in lipid biosynthesis and inflammatory response [15–17, 42], it is not surprising to find the associations of CD5L with HCC [20, 21, 23, 43, 44]. As liver plays critical roles in numerous biological processes [25], we speculated that CD5L dysregulation in HCC might have multiple roles during the tumor development and progression. Here, through CD5L-associated gene analysis in HCC, we identified 14 biological pathways which CD5L might be involved in. Besides lipid metabolism (fatty acid metabolism and bile acid metabolism) and immune processes (interferon gamma response, interferon alpha response, allograft rejection, IL-6 JAK stat3 signaling, inflammatory response and complement), xenobiotic metabolism, fatty acid coagulation, MYC targets v1, mitotic spindle, G2M checkpoint, and E2F targets were also included, indicating the multiple potentials of CD5L in HCC. Consistently, in the subsequent analyses, CD5L was also found to be correlated immunoregulators and immune cell infiltrations, consistent with the immune pathways in the GSEA results. With systemic analyses, we identified 28 CD5L-APDGs. Among them, LCAT and CDC20 were highlighted for their independent prognostic effects and dysregulations in HCC. Furthermore, with the two genes, a risk model for HCC OS was constructed. The risk model could discriminate the HCC OS status efficiently in both the training set and the validation set, indicating its robustness in the predication of HCC prognosis. Their associations with HCC immune response and anti-cancer drug sensitivity uncovered their potential values in HCC immunoregulation and drug therapy. In addition, the protein-chemical interactions of the three proteins were analyzed and more than 90 chemicals were shown to be associated with their dysregulations, providing new clues for HCC prevention.

LCAT gene encodes lecithin-cholesterol acyltransferase (LCAT), the only enzyme capable of esterifying cholesterol in plasma and help transport excess cholesterol to liver from the blood and tissues [45–47]. As LCAT and CD5L were important for lipid metabolism, it’s not surprising to find their correlations. The crucial roles of LCAT in cholesterol metabolism were also in accordance with fatty acid metabolism in CD5L-associated pathways. In previous studies, the reduction of LCAT activity was shown to be associated with atherosclerosis [48, 49]. LCAT dysregulation was also shown in many malignancies. In breast cancer, LCAT overexpression was demonstrated to be associated with the tumor grade and aggressiveness [50]. In contrast, its decrease was shown in colorectal cancer [51] and ovarian cancer [52]. In HCC, the decrease of LCAT was reported and its prognostic effects were shown in several studies [37, 53–55]. Here, we detected serum LCAT activity in HCC and found its lower level. In a previous study, it was reported that the adenovirus-mediated transfer of human LCAT gene could lead to the increase of HDL [56]. Here, consistently, the positive correlation of serum LCAT activity with HDL concentration was also shown, indicating the dysregulation of cholesterol in HCC. We also found the downregulation and prognostic roles of LCAT expression in HCC, as well as its significant correlations with CD5L expression and CDC20 expression. As one of CD5L-correlated genes, its correlations with immune response in HCC were also shown, indicating the roles of cholesterol metabolism in HCC immunomodulation. Their downregulation, prognostic effects and their positive correlation indicated that they might be involved in similar pathways during HCC development and progression.

CDC20 gene encodes cell-division cycle protein 20 homologue (CDC20), a protein which is crucial for chromosome segregation and mitotic exit [57]. It could regulate cell cycle progression via targeting its key substrates containing a destruction-box (D-box) for destruction [58]. In this study, the cell cycle related processes were also included in the GSEA results, consistent with the functions of CDC20 and its negative correlations with CD5L with HCC. The tumor-promoting activities of CDC20 were reported in many tumors including pancreatic ductal adenocarcinoma [59], lung adenocarcinoma [60], gastric cancer [61], breast cancer [62, 63] and bladder cancer [64]. Here, the upregulation and unfavorable prognostic effects of CDC20 were shown in HCC, consistent with previous studies [65–69]. Notably, comparing the discriminating power (AUC: 0.621) of the four-gene signature (BRCA1-CAD-CDC20-RBM8A) in Wang study [70], with an AUC of 0.656 in TCGA-HCC dataset and 0.740 in ICGC-HCC dataset, the superior performance of the two-gene combination (LCAT-CDC20) in this study was obvious. Furthermore, the negative correlations between LCAT and CDC20 were shown for at both mRNA and protein levels, indicating the associations between cholesterol metabolism and cell cycle process. The immunoregulatory roles of cyclin-dependent kinases in previous studies [71, 72]. In this study, the associations of CDC20 with immunoregulatory genes were shown, further confirmed the close links between cell cycle processes and immune regulation.

The pivotal roles of tumor microenvironment in tumor occurrence and progression were confirmed in numerous studies. In HCC, immune evasion was demonstrated to be one of the factors which could lead to its low response rate to the immunotherapies [73, 74]. CD5L is mainly expressed by tissue macrophages [75]. Here, the lower CD5L expression in HCC tissues (stroma) might be due to the lower infiltrations of macrophages in HCC. As liver is not the only source of CD5L, it is not surprising to see the inconsistence between HCC CD5L and serum CD5L. It was reported that resident and recruited macrophages in liver are the key parts for its homeostatic function and response to tissue damage [76], the lower level of macrophages in HCC might be associated with HCC development. However, although the positive correlations of CD5L with macrophages were obvious in this study, none of the macrophages presented significant independent prognostic effects on HCC OS. In contrast, the infiltrations of adipocytes (stroma cell), lymphatic endothelial cells (stroma cell), and the CLP cells [a kind of hematopoietic stem cells (HSCs)] were shown to have prognostic effects on HCC OS. Interestingly, all of the three kinds of cells presented significant correlations with CD5L, LCAT and CDC20 expressions in HCC tumors. As important parts of tumor microenvironment, the tumor stroma was demonstrated to play important roles in tumor progression and accounted for the poor prognosis of many malignancies including lung cancer [77], breast cancer [78], colorectal cancer [79] and gastric adenocarcinoma [80]. In this study, all the three genes presented significant correlations with stroma score in HCC, indicating their involvements in the regulation of HCC stroma.

Pro-B is an early stage of B-cell development [81] and the immunoregulatory properties of innate pro-B cells were demonstrated in a previous study [82]. Here, the unfavorable prognostic effects of pro-B cells on HCC OS were shown and the positive correlation of CDC20 with pro-B cell infiltration were obvious. Based on the overexpression of CDC20 in the tumors, we speculated that there might be a regulatory potential of CDC20 dysregulation in B cell response in HCC. Th2 cells are important parts of the tumor microenvironment and have been found to be associated with tumor development and progression [83]. In breast cancer [84], high levels of Th2 cell infiltration were also reported to be associated with poor prognosis of the patients. In cervical carcinoma [85], Th2 cells were demonstrated to be associated with the tumor progression. In colorectal cancer [86], Th2 cells were found to be associated with the metastasis of the tumors. In this study, Th2 cell infiltration was also indicated to be another prognostic indicator for HCC OS, indicating its crucial roles in HCC progression. We also found the negative correlation of LCAT while positive correlation of CDC20 with Th2 cell infiltration which indicated their regulatory potential in HCC immune response. In addition, the significant correlations of CD5L, LCAT, and CDC20 with immunoregulators were shown. PD-1 and PD-L1 were two components in the programmed death-1 pathway and they were reported to be upregulated in the tumor microenvironment and could lead to immune suppression and tumor immune escape [87]. In many cancerous diseases [88–91], their inhibitors were reported to be effective to prolong the OS of the patients to some extent. Although many PD-1/L1 inhibitors have been approved for the treatment of advanced HCC, the efficiency is not satisfactory due to the heterogeneity of the patients [92–94]. CTLA-4 was expressed exclusively on T cells and its blockade could result in the enhancement of immune responses dependent on helper T cells [95]. In this study, we uncovered the significant correlations of CD5L, LCAT and CDC20 with immunoregulators including PD-1/L1 and/or CTLA4 and the results might provide new clues for HCC immunotherapy.

With its strict association with cell proliferation, MKI67 is often used as a proliferation marker [33]. Here, the negative correlation of LCAT while the positive correlation of CDC20 with MKI67 in HCC were shown, indicating their associations with HCC growth. However, no significant correlation between CD5L and MKI67 was shown, inconsistent with their positive correlation reported in a previous study [24]. In addition, in this study, CD5L was shown to be down-regulated at both mRNA and protein levels, opposite to the upregulation in Aran study [24]. One explanation for the inconsistence of these results was the sample size [96]. Notably, in this study, HCC 159 pairs of HCC tumors (n = 159) and normal liver controls (n = 159) were used for analyses of the proteins, almost three times of the Aran study (60 HCCs and 34 nontumor livers). Another explanation might be the heterogeneity between the samples of different studies. In our previous study, CD5L expression was negatively correlated the tumor stage of HCC and its prognostic effect was not independent of the tumor stage [23]. The opposite results of the prognostic effects of CD5L at mRNA level and protein level through Kaplan–Meier analysis might be due to the difference between its correlated genes and its correlated proteins in the samples. The unfavorable prognostic effects of CD5L expression at protein level might be explained by its positive correlation with serum AFP. In addition, CD5L functions as a secretory protein. As no significant difference of serum CD5L was shown between normal and HCC samples, we speculated that there might be some difference between serum CD5L and tissue CD5L in HCC samples. The important roles of post-translational modifications have been reported in plenty of studies and there might be modified CD5L in HCC tissues which might be associated with its prognostic effect. However, considering the anti-tumor activities in HCC [20, 21, 97] and its pleiotropic functions in liver diseases [98], further study is need to investigate the specific roles of CD5L in HCC.

In this study, we also explored the potential roles of CD5L, LCAT, and CDC20 in HCC therapy and found their associations with anti-cancer drug sensitivities in HCC cell lines. These results might provide clues for the study of drug-resistance in HCC and HCC treatment. In addition, from the protein-chemical interaction analysis, more than 80 chemicals were found to be associated with at least one of CD5L, LCAT, and CDC20. Among them, aflatoxin B1 and benzo(a)pyrene were highlighted for their interactions with all of the three proteins. Similar to the dysregulations of the three proteins in HCC and the immune associations, the tumor-promoting roles of aflatoxin B1 and Benzo(a)pyrene in HCC development and progression were also reported in many studies [39, 99–101] and their associations with HCC immune response[101] were shown. These protein-chemical interactions would provide new clues for HCC etiology and mechanism studies.

## Conclusion

In summary, besides lipid metabolism and immune related pathways, CD5L might be associated with xenobiotic metabolism, coagulation and cell cycle related processes, indicating its multiple roles in HCC. The 28 CD5L-APDGs with prognostic effects and dysregulations in HCC could provide new clues for further study of the mechanism of HCC progression. LCAT was downregulated while CDC20 was upregulated in HCC. The LCAT-CDC20 signature might be a new marker for HCC prognosis. Serum LCAT activity was lower in HCC patients and might be a new diagnostic maker for HCC. LCAT and CDC20 were associated with HCC microenvironment and proliferation. They might be effective markers for HCC diagnosis and progression as well as new targets for HCC therapy. However, considering the complexity in the processes of gene transcription and translation, further study is needed to investigate the mechanisms of the dysregulations of CD5L, LCAT and CDC20.

## Supplementary Information

**Additional file 1: Table S1.** Clinical characters of the patients in TCGA-HCC dataset and ICGC-HCC dataset. **Table S2.** The clinical characters of HCC patients from Clinical Proteomic Tumor Analysis Consortium (CPTAC). **Table S3**. The gender-age-stage-corrected prognostic effects of the 256 CD5L-AGs in HCC. **Table S4.** The dysregulations of 28 CD5L-APDGs in TCGA-HCC and ICGC-HCC datasets. **Table S5.** The comparisons of the immune/stroma infiltrations between HCC tissues and liver controls. **Table S6.** The correlations of CD5L, LCAT and CDC20 with the infiltrations of the immune and stroma cells in HCC. **Table S7.** The prognostic effects of the immune and stroma cells correlated with CD5L, LCAT, and/or CDC20 in HCC. **Figure S1.** Prognostic effects of CD5L, LCAT and CDC20 on HCC OS. (A-B) Significant favorable prognostic effects of CD5L and LCAT on HCC OS in TCGA-HCC dataset. (C) Significant unfavorable prognostic effects of CDC20 on HCC OS in TCGA-HCC dataset. (D-E) Significant favorable prognostic effects of CD5L and LCAT on HCC OS in ICGC-HCC dataset. (F) Significant unfavorable prognostic effects of CDC20 on HCC OS in ICGC-HCC dataset. OS, overall survival; HCC, hepatocellular carcinoma. Kaplan-Meier survival analysis was used and *p* < 0.05 was considered significant. **Figure S2.** The correlations of CD5L, LCAT and CDC20 expressions with NK-κB associated genes. **Figure S3**. The correlations of CD5L, LCAT, and CDC20 with chemokines and chemokine receptors in HCC. **Figure S4.** Tumor stage proportion comparisons between different HCC datasets. **Figure S5**. CD5L, LCAT and CDC20 expression comparisons between CPTAC-HCC samples of different stages and normal livers. **Figure S6.** Correlations of tissue AFP and ALB with serum AFP and ALB in HCC. **Figure S7**. Subcellular location of CD5L, LCAT and CDC20 in human protein atlas (HPA). **Figure S8.** Immunohistochemistry staining of CD5L and CDC20 in HCC and liver tissues.

**Additional file 2. **The correlations of the three genes with anti-cancer drug sensitivities in HCC cell lines.

**Additional file 3. **ELISA detection of serum CD5L, LCAT, AFP, ALB, and HDL.

## Data Availability

The datasets presented in this study could be found in online database. The original contributions presented in the study are deposited in the article and Additional files, further inquiries can be directed to the corresponding author.

## Abbreviations

HCC: Hepatocellular carcinoma
CD5L-AGs: CD5L-associated genes
GSEA: Gene set enrichment analysis
LASSO: Least absolute shrinkage and selection operator
CPTAC: Clinical proteomic tumor analysis consortium
CD5L-APDGs: CD5L-associated prognostic and diagnostic genes
AIM: Apoptosis inhibitor of macrophages
TCGA: The Cancer Genome Atlas
ICGC: International cancer genome consortium
CCLE: Cancer cell line encyclopedia
LN_IC50s: Log-transformed half maximal inhibitory concentrations
HPA: Human Protein Atlas
AFP: Alpha-fetoprotein
ALB: Albumin
HDL: High density lipoprotein
