# Supplementary material for: CD5L-associated gene analyses highlight the dysregulations, prognostic effects, immune associations, and drug-sensitivity predicative potentials of LCAT and CDC20 in hepatocellular carcinoma
Source: Cancer Cell Int. 2022 Dec 9;22:393. doi: 10.1186/s12935-022-02820-7 (PMC9733014; doi:10.1186/s12935-022-02820-7)
Supplement: Supplementary file 1 — Additional file 1: Table S1. Clinical characters of the patients in TCGA-HCC dataset and ICGC-HCC dataset. Table S2. The clinical characters of HCC patients from Clinical Proteomic Tumor Analysis Consortium (CPTAC). Table S3. The gender-age-stage-corrected prognostic effects of the 256 CD5L-AGs in HCC. Table S4. The dysregulations of 28 CD5L-APDGs in TCGA-HCC and ICGC-HCC datasets. Table S5. The comparisons of the immune/stroma infiltrations between HCC tissues and liver controls. Table S6. The correlations of CD5L, LCAT and CDC20 with the infiltrations of the immune and stroma cells in HCC. Table S7. The prognostic effects of the immune and stroma cells correlated with CD5L, LCAT, and/or CDC20 in HCC. Figure S1. Prognostic effects of CD5L, LCAT and CDC20 on HCC OS. (A-B) Significant favorable prognostic effects of CD5L and LCAT on HCC OS in TCGA-HCC dataset. (C) Significant unfavorable prognostic effects of CDC20 on HCC OS in TCGA-HCC dataset. (D-E) Significant favorable prognostic effects of CD5L and LCAT on HCC OS in ICGC-HCC dataset. (F) Significant unfavorable prognostic effects of CDC20 on HCC OS in ICGC-HCC dataset. OS, overall survival; HCC, hepatocellular carcinoma. Kaplan-Meier survival analysis was used and p < 0.05 was considered significant. Figure S2. The correlations of CD5L, LCAT and CDC20 expressions with NK-κB associated genes. Figure S3. The correlations of CD5L, LCAT, and CDC20 with chemokines and chemokine receptors in HCC. Figure S4. Tumor stage proportion comparisons between different HCC datasets. Figure S5. CD5L, LCAT and CDC20 expression comparisons between CPTAC-HCC samples of different stages and normal livers. Figure S6. Correlations of tissue AFP and ALB with serum AFP and ALB in HCC. Figure S7. Subcellular location of CD5L, LCAT and CDC20 in human protein atlas (HPA). Figure S8. Immunohistochemistry staining of CD5L and CDC20 in HCC and liver tissues. [file 12935_2022_2820_MOESM1_ESM.pdf]

Supplementary files

Table S1. Clinical characters of the patients in TCGA-HCC dataset and ICGC-HCC dataset.

Variables	TCGA-HCC (n=371)	ICGC-HCC (n=223)
Gender		
Male / female	250 / 121	164 / 59
Age (year)		
<60 / 60-70 / >70 / NA	169 / 128 / 73 / 1	43 / 80 / 100 / 0
TNM stage		
I/ II/ III/ IV/ NA	171 / 86 / 85 / 5 / 24	36 / 102 / 67 /18 /0
Survival status		
Alive / dead / NA	240 / 130 / 1	181 / 42 / 0

HCC, hepatocellular carcinoma; TCGA, The Cancer Genome Atlas; ICGC, International CancerGenome Consortium; NA, not available.

Table S2. The clinical characters of HCC patients from Clinical Proteomic Tumor Analysis Consortium (CPTAC).

Variables	CPTAC-HCC (n=159)
Gender	
Male / Female	128 / 31
Age (year)	
<60 / 60-70 / >70	108 / 42 / 9
Tumor size (cm)	
<=5 / >5	77 / 82
Survival status	
Alive / Dead / NA	99 / 51 / 9

HCC, hepatocellular carcinoma; NA, not available.

Table S3. The gender-age-stage-corrected prognostic effects of the 256 CD5L-AGs on HCC OS.

		TCGA-HCC		ICGC-HCC	
		HR (95%CI)	*P* value	HR (95%CI)	*P* value
1	KLRK1	0.927(0.867-0.992)	0.0283*	0.781(0.642-0.95)	0.0135*
2	ADRA2B	0.849(0.727-0.992)	0.0396*	0.751(0.599-0.941)	0.0127*
3	ESR1	0.914(0.843-0.991)	0.0303*	0.7(0.584-0.84)	0.000121**
4	LCAT	0.847(0.752-0.953)	0.00599**	0.71(0.566-0.891)	0.00313**
5	ALDOA	1.22(1.04-1.42)	0.0131*	1.31(1.01-1.7)	0.0442*
6	DDX39A	1.38(1.11-1.71)	0.00335**	1.68(1.1-2.57)	0.0163*
7	CENPA	1.31(1.16-1.47)	7.31E-06**	1.75(1.38-2.22)	3.79E-06**
8	SAC3D1	1.45(1.18-1.78)	0.000329**	1.71(1.18-2.46)	0.00454**
9	TRAIP	1.23(1.07-1.42)	0.00292**	1.68(1.25-2.25)	0.000517**
10	TIPIN	1.44(1.14-1.82)	0.00193**	2.28(1.52-3.42)	6.42E-05**
11	RNASEH2A	1.31(1.11-1.55)	0.00177**	1.96(1.34-2.86)	0.000473**
12	DTYMK	1.5(1.23-1.83)	6.70E-05**	2.18(1.52-3.13)	2.36E-05**
13	CDC20	1.29(1.16-1.44)	5.71E-06**	1.56(1.25-1.93)	7.09E-05**
14	BAK1	1.27(1.08-1.5)	0.00496**	1.47(1.08-2)	0.0137*
15	MCM3	1.29(1.09-1.54)	0.00372**	1.68(1.21-2.33)	0.002**
16	TUBG1	1.68(1.33-2.12)	1.10E-05**	2.46(1.58-3.82)	6.14E-05**
17	HES6	1.22(1.02-1.46)	0.0279*	1.33(1.05-1.69)	0.0203*
18	CDCA8	1.38(1.21-1.58)	2.47E-06**	1.8(1.38-2.33)	1.05E-05**
19	PPIA	1.82(1.31-2.54)	0.000411**	3.53(1.94-6.43)	3.51E-05**
20	UBE2C	1.25(1.12-1.4)	0.000123**	1.68(1.33-2.12)	1.32E-05**
21	BIRC5	1.25(1.11-1.4)	0.000161**	1.69(1.35-2.11)	4.73E-06**
22	SPC24	1.18(1.04-1.35)	0.0134*	1.45(1.1-1.92)	0.00863**
23	KIF2C	1.3(1.16-1.46)	1.06E-05**	1.71(1.34-2.19)	2.04E-05**
24	CDC6	1.22(1.08-1.38)	0.00152**	1.72(1.35-2.18)	8.75E-06**
25	UBE2T	1.3(1.12-1.52)	0.000663**	1.88(1.38-2.57)	6.48E-05**
26	RBL1	1.31(1.12-1.55)	0.00114**	1.52(1.09-2.11)	0.0132*
27	SPC25	1.33(1.16-1.53)	3.40E-05**	2(1.49-2.7)	4.83E-06**
28	TROAP	1.23(1.1-1.37)	0.000375**	1.79(1.38-2.34)	1.69E-05**
29	SLBP	1.51(1.16-1.96)	0.00199**	2.11(1.31-3.39)	0.00216**
30	RFC4	1.37(1.15-1.63)	0.000514**	1.74(1.27-2.38)	0.000503**
31	MYBL2	1.18(1.08-1.29)	0.000226**	1.55(1.29-1.87)	3.32E-06**
32	CDCA3	1.3(1.14-1.48)	1.00E-04**	1.85(1.44-2.38)	1.41E-06**
33	TACC3	1.28(1.11-1.47)	0.00088**	1.69(1.26-2.29)	0.000559**
34	HMGA1	1.3(1.13-1.49)	0.000194**	1.45(1.14-1.84)	0.00281**
35	CENPF	1.22(1.09-1.37)	0.00074**	1.58(1.23-2.04)	0.000367**
36	ASF1B	1.21(1.06-1.37)	0.00337**	1.66(1.3-2.13)	6.22E-05**
37	CENPM	1.22(1.09-1.36)	0.000629**	1.55(1.24-1.93)	9.29E-05**
38	CCNF	1.32(1.13-1.55)	0.000576**	1.73(1.29-2.32)	0.000283**
39	PLK1	1.28(1.13-1.44)	5.81E-05**	1.57(1.23-2)	0.000259**
40	TCF19	1.19(1.05-1.35)	0.00621**	1.79(1.37-2.35)	2.61E-05**
41	PRC1	1.21(1.07-1.38)	0.00339**	2.14(1.57-2.9)	1.22E-06**
42	MELK	1.28(1.13-1.45)	0.000115**	1.73(1.33-2.24)	3.71E-05**
43	MKI67	1.24(1.1-1.4)	0.000372**	1.62(1.25-2.09)	0.000233**

44	KIF4A	1.23(1.1-1.38)	0.000258**	2.02(1.51-2.7)	2.07E-06**
45	EXO1	1.25(1.1-1.42)	0.000429**	1.82(1.37-2.41)	3.48E-05**
46	CDKN3	1.22(1.08-1.38)	0.00122**	1.77(1.35-2.32)	3.82E-05**
47	GINS1	1.29(1.14-1.47)	6.70E-05**	1.73(1.33-2.25)	4.11E-05**
48	KIF23	1.2(1.08-1.35)	0.00117**	1.76(1.37-2.27)	8.86E-06**
49	SNRPB	1.4(1.14-1.73)	0.00143**	2.19(1.42-3.37)	0.000365**
50	PRIM2	1.35(1.09-1.68)	0.00624**	2(1.33-3)	0.000852**
51	TOP2A	1.22(1.09-1.36)	0.000358**	1.64(1.29-2.1)	5.76E-05**
52	EZH2	1.44(1.22-1.71)	2.55E-05**	1.96(1.38-2.77)	0.000146**
53	EIF2S2	1.63(1.22-2.19)	0.00107**	2.2(1.25-3.86)	0.00614**
54	GINS2	1.18(1.02-1.37)	0.0249*	1.61(1.2-2.16)	0.0015**
55	ECT2	1.24(1.09-1.41)	0.000799**	1.49(1.16-1.9)	0.00152**
56	TPX2	1.35(1.18-1.55)	1.17E-05**	1.94(1.47-2.56)	3.55E-06**
57	UBE2S	1.35(1.16-1.57)	0.000139**	1.7(1.24-2.33)	0.000879**
58	CCNB2	1.21(1.07-1.36)	0.0024**	1.51(1.18-1.94)	0.00101**
59	CDK1	1.27(1.12-1.43)	0.000154**	1.98(1.45-2.72)	2.14E-05**
60	RAD51C	1.49(1.16-1.9)	0.0015**	2.47(1.63-3.73)	1.89E-05**
61	PA2G4	1.83(1.34-2.5)	0.000133**	5.33(2.69-10.6)	1.65E-06**
62	CENPE	1.28(1.13-1.44)	0.00011**	1.71(1.24-2.34)	0.000898**
63	SNRPA1	1.57(1.21-2.02)	0.000573**	1.9(1.26-2.86)	0.00208**
64	E2F3	1.19(1.01-1.39)	0.0321*	1.5(1.01-2.23)	0.0421*
65	ORC6	1.25(1.1-1.42)	0.000593**	1.47(1.15-1.88)	0.00246**
66	PIF1	1.22(1.08-1.38)	0.00168**	1.45(1.15-1.84)	0.00178**
67	NEK2	1.23(1.09-1.37)	0.000441**	1.72(1.33-2.23)	3.60E-05**
68	AURKB	1.23(1.1-1.38)	0.000309**	1.63(1.29-2.06)	4.16E-05**
69	UCK2	1.68(1.36-2.08)	1.33E-06**	1.64(1.16-2.32)	0.00488**
70	DLGAP5	1.3(1.15-1.46)	1.85E-05**	1.63(1.26-2.11)	0.000189**
71	ANLN	1.27(1.13-1.42)	4.69E-05**	1.7(1.33-2.17)	1.88E-05**
72	MCM2	1.3(1.13-1.49)	0.000197**	1.74(1.34-2.26)	2.65E-05**
73	KIF11	1.3(1.13-1.49)	0.000213**	1.59(1.18-2.13)	0.00231**
74	RAC1	1.48(1.14-1.92)	0.00337**	1.87(1.13-3.1)	0.015*
75	BUB1	1.27(1.12-1.44)	0.000136**	1.82(1.38-2.41)	2.57E-05**
76	KIF18B	1.24(1.11-1.38)	0.000131**	1.47(1.18-1.82)	0.000451**
77	BUB1B	1.23(1.09-1.38)	0.00045**	1.77(1.36-2.31)	1.94E-05**
78	E2F1	1.2(1.07-1.36)	0.00259**	1.62(1.3-2.03)	2.14E-05**
79	STIL	1.19(1.04-1.37)	0.0126*	1.45(1.07-1.96)	0.0152*
80	NDC80	1.4(1.21-1.61)	3.25E-06**	2.11(1.53-2.91)	6.22E-06**
81	ERP29	1.58(1.16-2.16)	0.00396**	2.95(1.61-5.39)	0.000457**
82	CCT3	1.73(1.34-2.22)	1.98E-05**	1.8(1.09-2.95)	0.0206*
83	PPM1G	1.96(1.44-2.67)	1.67E-05**	2.95(1.68-5.15)	0.000153**
84	NCBP2	1.73(1.31-2.28)	0.000106**	1.65(1.02-2.68)	0.0429*
85	CFP	0.974(0.852-1.12)	0.707	0.792(0.63-0.996)	0.0461*
86	FCN1	1(0.885-1.14)	0.951	0.852(0.679-1.07)	0.169
87	PRKCB	0.99(0.877-1.12)	0.872	0.72(0.564-0.919)	0.00824**
88	KLRD1	0.935(0.821-1.06)	0.307	0.722(0.565-0.924)	0.00962**
89	CCL24	1.01(0.951-1.08)	0.704	0.944(0.858-1.04)	0.241
90	CR1	1.02(0.935-1.11)	0.681	0.853(0.693-1.05)	0.134

91	FGL2	0.975(0.853-1.12)	0.716	0.793(0.625-1.01)	0.0556
92	PRF1	0.925(0.806-1.06)	0.265	0.798(0.63-1.01)	0.0634
93	MARCO	1.02(0.954-1.08)	0.63	0.968(0.878-1.07)	0.505
94	CCL4	1.02(0.896-1.15)	0.805	0.838(0.672-1.05)	0.117
95	GZMK	0.964(0.884-1.05)	0.414	0.829(0.703-0.978)	0.0259*
96	IL10RA	0.98(0.847-1.13)	0.788	0.807(0.628-1.04)	0.0922
97	C1QC	1.05(0.92-1.21)	0.453	0.88(0.715-1.08)	0.225
98	CD38	1.04(0.935-1.15)	0.509	0.865(0.722-1.04)	0.115
99	ITK	0.934(0.842-1.04)	0.202	0.812(0.7-0.942)	0.00595**
100	C1QA	1.02(0.894-1.17)	0.747	0.842(0.678-1.04)	0.118
101	CD247	0.949(0.819-1.1)	0.486	0.76(0.601-0.961)	0.0218*
102	CD8A	0.925(0.841-1.02)	0.108	0.848(0.708-1.02)	0.0727
103	IGSF6	1.06(0.909-1.23)	0.468	0.873(0.687-1.11)	0.269
104	SELE	0.986(0.913-1.06)	0.713	0.88(0.765-1.01)	0.072
105	CXCL9	0.975(0.895-1.06)	0.568	0.884(0.763-1.02)	0.101
106	CXCR6	0.945(0.858-1.04)	0.255	0.951(0.818-1.11)	0.514
107	CD69	0.99(0.907-1.08)	0.814	0.826(0.723-0.942)	0.00455**
108	ZAP70	0.948(0.833-1.08)	0.416	0.752(0.616-0.919)	0.00526**
109	CD4	0.953(0.822-1.1)	0.523	0.731(0.553-0.965)	0.0271*
110	MT2A	0.974(0.881-1.08)	0.606	0.856(0.723-1.01)	0.0707
111	LAP3	1.05(0.866-1.27)	0.638	0.919(0.664-1.27)	0.608
112	KCNA3	0.969(0.905-1.04)	0.381	0.891(0.781-1.02)	0.0839
113	CCR1	1.04(0.909-1.18)	0.603	0.851(0.674-1.07)	0.175
114	IL2RB	0.992(0.88-1.12)	0.89	0.805(0.643-1.01)	0.0566
115	ABCA8	0.97(0.898-1.05)	0.441	0.854(0.739-0.986)	0.031*
116	PTPRC	0.973(0.875-1.08)	0.607	0.822(0.667-1.01)	0.0668
117	ACSL5	1.03(0.909-1.16)	0.686	1.02(0.82-1.26)	0.889
118	SLAMF7	0.986(0.891-1.09)	0.782	0.897(0.754-1.07)	0.22
119	IL7R	0.964(0.884-1.05)	0.399	0.893(0.786-1.01)	0.0827
120	AQP9	0.961(0.9-1.03)	0.234	0.901(0.802-1.01)	0.077
121	CYP27A1	0.926(0.809-1.06)	0.266	0.829(0.673-1.02)	0.0774
122	FASLG	0.981(0.896-1.07)	0.68	0.892(0.773-1.03)	0.114
123	MTHFD1	0.919(0.793-1.06)	0.261	0.788(0.6-1.04)	0.0875
124	PTGER2	0.959(0.862-1.07)	0.435	0.81(0.689-0.951)	0.00994**
125	SLC6A12	0.987(0.897-1.09)	0.792	0.999(0.831-1.2)	0.991
126	TRAT1	0.973(0.905-1.05)	0.461	0.933(0.844-1.03)	0.176
127	CYB5A	0.986(0.84-1.16)	0.867	0.889(0.672-1.17)	0.406
128	GLUL	1.01(0.927-1.11)	0.753	0.972(0.812-1.16)	0.756
129	SLC1A2	0.972(0.912-1.04)	0.376	1.04(0.922-1.17)	0.522
130	RGN	0.917(0.815-1.03)	0.151	0.941(0.787-1.13)	0.507
131	CD36	1.01(0.898-1.12)	0.93	1.07(0.894-1.28)	0.455
132	ENPEP	0.971(0.861-1.09)	0.629	0.836(0.681-1.03)	0.0885
133	PC	1.11(0.944-1.32)	0.199	0.944(0.708-1.26)	0.697
134	C1S	0.847(0.726-0.989)	0.0351*	0.843(0.664-1.07)	0.16
135	C1R	0.859(0.729-1.01)	0.068	0.682(0.523-0.889)	0.00466**
136	ALAS1	0.879(0.741-1.04)	0.139	0.83(0.628-1.1)	0.189
137	CDO1	0.981(0.904-1.06)	0.64	0.903(0.813-1)	0.0574

138	SLC27A5	0.922(0.841-1.01)	0.0839	0.858(0.742-0.992)	0.0388*
139	MCEE	0.859(0.678-1.09)	0.211	0.669(0.374-1.2)	0.177
140	CYP2E1	1.01(0.962-1.05)	0.82	0.967(0.892-1.05)	0.426
141	PLG	0.962(0.887-1.04)	0.356	0.978(0.881-1.09)	0.678
142	NCR1	0.948(0.886-1.01)	0.125	0.968(0.877-1.07)	0.522
143	ANGPTL3	1.04(0.947-1.13)	0.442	0.924(0.823-1.04)	0.184
144	VCAM1	1.07(0.97-1.17)	0.186	0.858(0.715-1.03)	0.0998
145	HSD17B4	1.01(0.847-1.19)	0.954	0.674(0.488-0.931)	0.0167
146	AOX1	0.945(0.877-1.02)	0.146	0.958(0.848-1.08)	0.496
147	CYP2J2	1.01(0.9-1.14)	0.822	0.909(0.758-1.09)	0.303
148	IRF4	0.982(0.905-1.06)	0.655	0.926(0.814-1.05)	0.247
149	IFNG	1(0.949-1.06)	0.964	0.984(0.869-1.11)	0.798
150	LEPR	1.03(0.948-1.12)	0.478	1(0.854-1.18)	0.983
151	ACAA1	1(0.826-1.21)	0.996	0.756(0.517-1.11)	0.15
152	MASP2	0.962(0.894-1.04)	0.311	0.877(0.775-0.993)	0.0379
153	SERPING1	0.864(0.727-1.03)	0.0954	0.548(0.429-0.699)	1.38E-06**
154	SERPINC1	0.977(0.909-1.05)	0.517	0.95(0.863-1.05)	0.291
155	IFIT3	1.04(0.888-1.23)	0.598	0.677(0.505-0.907)	0.00895**
156	ATOH8	0.924(0.845-1.01)	0.087	0.762(0.663-0.875)	0.000118**
157	GRHPR	0.875(0.72-1.06)	0.181	0.619(0.441-0.869)	0.00563**
158	AKR1D1	0.966(0.911-1.02)	0.245	0.93(0.84-1.03)	0.161
159	C8A	0.929(0.853-1.01)	0.094	0.939(0.831-1.06)	0.318
160	ALDH2	0.771(0.639-0.93)	0.00662**	0.791(0.613-1.02)	0.0718
161	MBL2	0.98(0.912-1.05)	0.575	0.941(0.856-1.04)	0.211
162	AR	0.943(0.869-1.02)	0.158	0.953(0.872-1.04)	0.285
163	CYP8B1	0.97(0.922-1.02)	0.242	0.947(0.868-1.03)	0.216
164	GCDH	0.804(0.654-0.989)	0.0386*	0.833(0.574-1.21)	0.333
165	FMO3	0.952(0.88-1.03)	0.212	0.841(0.734-0.962)	0.0119*
166	EHHADH	0.909(0.814-1.02)	0.0914	0.887(0.742-1.06)	0.186
167	DMD	1.08(0.948-1.23)	0.25	0.82(0.643-1.05)	0.111
168	F12	0.969(0.893-1.05)	0.452	0.914(0.783-1.07)	0.253
169	CYP1A2	1(0.967-1.04)	0.854	0.912(0.843-0.986)	0.0215*
170	CYP1A1	1.03(0.981-1.08)	0.235	0.971(0.89-1.06)	0.513
171	ACOX2	0.984(0.867-1.12)	0.804	0.943(0.78-1.14)	0.548
172	SULT1B1	0.968(0.894-1.05)	0.43	0.965(0.873-1.07)	0.478
173	EPHX2	0.943(0.822-1.08)	0.403	0.779(0.618-0.984)	0.0358*
174	AMACR	0.969(0.872-1.08)	0.553	1.03(0.828-1.27)	0.808
175	HAO1	0.946(0.873-1.02)	0.169	0.951(0.857-1.06)	0.344
176	TTPA	0.986(0.906-1.07)	0.751	0.979(0.892-1.07)	0.652
177	POR	0.935(0.769-1.14)	0.5	0.965(0.713-1.31)	0.816
178	CFI	0.943(0.818-1.09)	0.417	0.827(0.663-1.03)	0.0904
179	HSDL2	1.01(0.811-1.27)	0.905	0.961(0.697-1.33)	0.81
180	RBP4	0.903(0.815-1)	0.0532	0.95(0.848-1.06)	0.37
181	UPB1	0.934(0.869-1.01)	0.0683	0.905(0.801-1.02)	0.111
182	G6PC	0.94(0.88-1)	0.0684	0.965(0.879-1.06)	0.463
183	IFIT1	0.994(0.874-1.13)	0.927	0.721(0.581-0.893)	0.00282**
184	LEAP2	0.946(0.857-1.04)	0.262	0.867(0.738-1.02)	0.0829

185	F9	0.986(0.931-1.04)	0.619	0.914(0.828-1.01)	0.0751
186	ABCA6	0.939(0.858-1.03)	0.177	0.828(0.695-0.987)	0.0353
187	ABHD6	1(0.845-1.19)	0.979	0.904(0.666-1.23)	0.519
188	GOT2	0.675(0.54-0.843)	0.000544**	0.797(0.574-1.11)	0.176
189	ABCA9	0.935(0.842-1.04)	0.211	0.672(0.551-0.82)	9.33E-05**
190	SLC23A2	0.984(0.853-1.14)	0.829	0.856(0.641-1.14)	0.291
191	FGA	0.949(0.868-1.04)	0.244	0.979(0.85-1.13)	0.767
192	APOL6	0.886(0.743-1.06)	0.181	0.613(0.458-0.821)	0.00101**
193	CAT	0.909(0.77-1.07)	0.26	0.846(0.644-1.11)	0.23
194	CA4	0.987(0.934-1.04)	0.64	0.938(0.859-1.03)	0.159
195	MLYCD	0.864(0.695-1.07)	0.19	0.566(0.37-0.867)	0.00882**
196	DCXR	0.932(0.824-1.05)	0.259	0.822(0.667-1.01)	0.0643
197	CYP4A22	0.969(0.902-1.04)	0.39	0.964(0.882-1.05)	0.422
198	CYP4A11	0.96(0.892-1.03)	0.267	0.905(0.807-1.02)	0.0883
199	PROC	0.974(0.867-1.09)	0.655	0.894(0.758-1.05)	0.185
200	XAF1	0.955(0.867-1.05)	0.36	0.715(0.56-0.913)	0.00718**
201	APOA1	0.963(0.898-1.03)	0.293	0.949(0.855-1.05)	0.316
202	ACSM3	0.996(0.904-1.1)	0.943	0.984(0.758-1.28)	0.906
203	RFC2	1.26(0.993-1.59)	0.0569	2.11(1.3-3.43)	0.00257**
204	TFRC	1.14(0.958-1.36)	0.139	1.79(1.26-2.55)	0.00112**
205	RAP1GAP	1.16(1.03-1.3)	0.0149*	1.05(0.867-1.28)	0.598
206	CLEC5A	1.1(1.03-1.18)	0.00634**	1.02(0.912-1.15)	0.709
207	CASP6	1.28(0.978-1.67)	0.0726	0.93(0.508-1.7)	0.815
208	P2RX4	1.26(1.05-1.51)	0.0119*	1.41(0.928-2.14)	0.107
209	POP7	1.3(1.02-1.67)	0.0375*	1.62(0.95-2.77)	0.0763
210	UPP1	1.17(0.981-1.4)	0.08	1.1(0.808-1.49)	0.548
211	BIN1	1.15(0.952-1.4)	0.147	1.34(0.87-2.07)	0.183
212	RBCK1	1.22(0.975-1.53)	0.082	1.18(0.746-1.87)	0.478
213	PRDX5	1.36(0.999-1.85)	0.0511	1.59(0.861-2.94)	0.138
214	IL11	1.06(0.996-1.13)	0.0653	0.953(0.867-1.05)	0.318
215	SNRPA	1.51(1.19-1.91)	0.000675**	1.34(0.845-2.13)	0.214
216	TRAF2	1.4(1.12-1.75)	0.00331**	1.26(0.836-1.91)	0.268
217	SLC35B2	1.26(0.987-1.61)	0.063	1.54(1.01-2.36)	0.0451*
218	FGD6	1.09(0.949-1.26)	0.216	0.841(0.646-1.1)	0.2
219	ITGA5	1.2(1.02-1.42)	0.0284*	0.851(0.603-1.2)	0.358
220	PSMD8	1.29(0.903-1.84)	0.161	1.39(0.728-2.64)	0.321
221	SLC25A3	1.35(0.956-1.91)	0.0885	3.13(1.61-6.11)	0.000814**
222	TUBA4A	1.21(1.04-1.41)	0.0137*	1.03(0.784-1.34)	0.853
223	OPHN1	1.07(0.947-1.22)	0.268	1.14(0.752-1.73)	0.537
224	IL10RB	1.39(1.04-1.85)	0.0281*	0.702(0.436-1.13)	0.144
225	RABIF	1.59(1.21-2.1)	0.00097**	1.35(0.788-2.32)	0.274
226	GNB2	1.19(0.92-1.54)	0.184	1.4(0.847-2.32)	0.188
227	MMP13	1.03(0.967-1.09)	0.409	1.05(0.971-1.13)	0.24
228	CTSD	1.25(0.985-1.58)	0.0668	0.865(0.594-1.26)	0.449
229	DCTPP1	1.24(0.998-1.55)	0.0525	1.56(1.06-2.28)	0.0242*
230	MAP1S	1.29(0.993-1.67)	0.0565	0.854(0.484-1.51)	0.586
231	SIRT6	1.46(1.13-1.89)	0.00404**	0.98(0.634-1.52)	0.928

232	DYRK2	1.2(1.01-1.42)	0.0403*	0.945(0.698-1.28)	0.711
233	ACOT8	1.54(1.13-2.1)	0.00689**	1.92(0.898-4.11)	0.0925
234	ADRM1	1.55(1.12-2.14)	0.00848**	1.91(0.939-3.89)	0.0742
235	CLIP2	1.01(0.898-1.13)	0.906	0.984(0.802-1.21)	0.88
236	SLC22A5	1.21(0.959-1.52)	0.109	0.927(0.608-1.41)	0.724
237	CHEK2	1.19(1.01-1.4)	0.0361*	1.26(0.895-1.76)	0.187
238	PSMC4	1.51(1.11-2.07)	0.00906**	1.44(0.814-2.56)	0.209
239	BPGM	1.25(0.963-1.62)	0.094	1.02(0.64-1.62)	0.935
240	NCAPH2	1.37(1.09-1.72)	0.00764**	1.3(0.754-2.26)	0.342
241	UBE2L3	1.75(1.29-2.37)	0.000322**	1.46(0.84-2.55)	0.178
242	KIF5B	1.28(1.02-1.62)	0.0363*	1.05(0.709-1.57)	0.794
243	ENO2	1.08(0.98-1.19)	0.119	0.96(0.813-1.13)	0.63
244	S100A10	1.3(1.13-1.51)	0.000382**	1.2(0.905-1.58)	0.21
245	SMOX	1.23(1.09-1.4)	0.00111**	1.08(0.88-1.32)	0.465
246	MMP11	1.03(0.942-1.13)	0.524	0.944(0.836-1.07)	0.351
247	GSS	1.53(1.11-2.1)	0.0101*	0.899(0.462-1.75)	0.755
248	SNRPD2	1.17(0.979-1.39)	0.0858	1.91(1.19-3.07)	0.00743**
249	CYTH2	1.37(1.06-1.77)	0.0165*	0.88(0.602-1.29)	0.509
250	GINS3	1.19(0.999-1.41)	0.0514	1.53(1.13-2.07)	0.00604**
251	SLC35B1	1.29(0.919-1.8)	0.142	2.53(1.25-5.1)	0.00971**
252	LGALS3	1.15(1.03-1.28)	0.0151*	0.923(0.78-1.09)	0.351
253	ITGB8	1.01(0.945-1.07)	0.852	0.965(0.883-1.05)	0.43
254	LONP1	1.59(1.13-2.22)	0.00764**	1.42(0.774-2.6)	0.259
255	HAX1	1.57(1.16-2.12)	0.00354**	1.59(0.866-2.9)	0.135
256	EFHC1	1.21(0.995-1.47)	0.0563	0.795(0.574-1.1)	0.169

*, $p < 0.05$; **, $p < 0.01$. OS, overall survival. Multi-variable Cox regression analysis was used and the age-gender-stage corrected prognostic effects of the genes were evaluated. The genes with significant prognostic effects ($p < 0.05$) consistently in the two datasets were considered significant. The genes with favorable prognostic effects were colored green while the ones with unfavorable prognostic effects were colored yellow.

Table S4. The dysregulations of 28 CD5L-APDGs in TCGA-HCC and ICGC-HCC datasets.

	TCGA-HCC (HCC vs. normal)		ICGC-HCC (HCC vs. normal)	
	LogFC	*p*.adj	LogFC	*p*.adj
LCAT	-2.64255	1.35E-25	-1.1179	1.72E-12
TRAIP	3.326562	1.90E-45	1.12637	1.68E-12
CDC20	4.6604	3.31E-47	1.607258	1.76E-11
CDCA8	3.651991	5.97E-43	1.350496	4.09E-11
UBE2C	4.575272	2.18E-47	1.690012	4.45E-12
BIRC5	4.526867	2.12E-49	1.470481	8.27E-12
KIF2C	4.240314	3.02E-45	1.409505	7.32E-12
UBE2T	3.888926	9.45E-59	1.441988	1.76E-12
SPC25	3.918095	2.04E-47	1.171564	2.53E-11
TROAP	4.635595	1.51E-49	1.584956	3.21E-12
CDCA3	3.719097	1.89E-46	1.511684	3.77E-12
CENPF	4.319513	3.45E-46	1.565475	3.30E-11
CENPM	4.024244	4.11E-43	1.44463	2.50E-11
TCF19	3.324744	6.89E-40	1.154859	5.99E-11
PRC1	3.732581	7.86E-46	1.318329	1.57E-11
MELK	4.730829	1.11E-52	1.477457	4.35E-11
EXO1	4.30229	1.13E-48	1.41011	4.96E-11
CDKN3	4.386422	3.11E-53	1.478656	2.48E-11
KIF23	3.742435	8.96E-36	1.235197	8.71E-11
TOP2A	4.519225	9.57E-45	1.512741	6.52E-11
CCNB2	4.375723	1.36E-47	1.514688	4.72E-12
CENPE	3.698753	1.52E-40	1.050606	4.99E-11
AURKB	3.852715	9.64E-43	1.465529	3.77E-12
ANLN	4.309091	9.03E-43	1.474968	4.56E-11
BUB1	4.129372	1.63E-44	1.327455	2.90E-11
BUB1B	4.204975	1.19E-40	1.432871	1.20E-11
E2F1	4.312773	5.72E-50	1.410752	1.76E-11
NDC80	3.882315	3.07E-49	1.219602	1.68E-11

CD5L-APDGs, CD5L-associated prognostic and diagnostic genes; LogFC, log_2foldchange.The gene comparisons were performed with “limma” package in R software and the adjusted p value (*p.adj*) < 1e-10 was considered significant.

Table S5. The comparisons of the immune/stroma infiltrations between HCC tissues and liver controls.

	TCGA-HCC			ICGC-HCC		
	p	Median(HCC)-median(normal)	Change	*p*	Median(HCC)-median(normal)	Change
aDC	5.699E-07	-0.06430727	down	1.50E-16	-0.101003761	down
Adipocytes	5.309E-15	-0.055663481	down	1.50E-39	-0.053869474	down
CD8+ Tcm	1.186E-07	-0.024856104	down	5.75E-23	-0.056396966	down
cDC	2.926E-09	-0.071876333	down	1.35E-20	-0.067935304	down
Fibroblasts	3.990E-14	-0.072088827	down	5.75E-18	-0.050797584	down
GMP	5.982E-25	-0.063757806	down	7.96E-58	-0.05905136	down
Hepatocytes	1.342E-29	-0.142386772	down	2.05E-58	-0.120861964	down
HSC	8.774E-24	-0.251580322	down	8.19E-43	-0.241409901	down
Macrophages	1.764E-16	-0.041102143	down	2.36E-14	-0.022356655	down
Macrophages M1	7.823E-11	-0.018056918	down	5.93E-11	-0.016854496	down
Macrophages M2	1.503E-10	-0.024879836	down	6.97E-14	-0.019652057	down
Megakaryocytes	3.062E-26	-0.020624289	down	2.05E-62	-0.020831373	down
MEP	4.253E-03	-0.007238957	down	0.025528631	-0.004663206	down
Monocytes	2.812E-20	-0.047509069	down	1.42E-35	-0.060064078	down
pDC	2.667E-06	-0.012424918	down	1.98E-06	-0.007701901	down
Pericytes	2.333E-22	-0.077109781	down	2.88E-52	-0.095976937	down
Preadipocytes	2.357E-06	-0.036973026	down	2.57E-06	-0.026346071	down
**ImmuneScore**	**3.108E-09**	**-0.053427501**	**down**	**2.93E-19**	**-0.070466669**	**down**
**StromaScore**	**7.422E-10**	**-0.047462795**	**down**	**1.46E-27**	**-0.039521758**	**down**
**MicroenvironmentScore**	**1.106E-10**	**-0.092827794**	**down**	**2.29E-26**	**-0.102618461**	**down**
Basophils	8.964E-04	0.023768819	up	4.71E-09	0.012461492	up
CLP	1.059E-22	0.015725369	up	4.00E-40	0.019875478	up
Mast cells	4.497E-15	0.003155589	up	5.51E-28	0.003247291	up
Melanocytes	5.544E-05	0.000998669	up	0.004977564	0.000409901	up
Neurons	8.117E-04	0.000259216	up	0.039237892	0.000141229	up
NKT	7.624E-13	0.013683808	up	3.00E-34	0.030122027	up
Osteoblast	2.517E-03	1.94E-18	up	6.95E-28	0.042931544	up
Plasma cells	6.099E-09	0.011391128	up	0.031449547	0.003660015	up
pro B-cells	1.260E-07	0.000681112	up	3.21E-21	0.013814303	up
Sebocytes	1.588E-06	0.001612359	up	5.88E-08	0.000869137	up
Th1 cells	2.244E-12	0.022880623	up	2.17E-14	0.024984215	up
Th2 cells	7.530E-15	0.032170812	up	2.18E-26	0.074523582	up
Astrocytes	2.599E-01	3.91E-19	stable	1.79E-08	2.29E-18	up
B-cells	6.225E-03	1.71E-18	up	0.51192589	1.14E-19	stable
CD4+ memory T-cells	1.260E-02	3.86E-19	up	0.780658783	0.001010547	stable
CD4+ naive T-cells	1.741E-01	-1.19E-19	stable	0.41141813	4.84E-19	stable
CD4+ T-cells	3.058E-01	4.68E-19	stable	0.066482095	-1.08E-18	stable
CD4+ Tcm	9.234E-06	1.11E-18	up	4.84E-06	-0.008252577	down
CD4+ Tem	6.727E-06	0.013332233	up	0.087182049	0.001201158	stable
CD8+ naive T-cells	2.280E-04	0.003333663	up	0.021895878	-0.001820146	down
CD8+ T-cells	1.316E-01	5.95E-18	stable	0.000191885	-0.011143138	down
CD8+ Tem	9.716E-01	5.00E-19	stable	6.80E-05	-0.011433593	down
Chondrocytes	7.822E-01	-2.20E-18	stable	0.444109082	6.76E-19	stable
Class-switched memory B-cells	4.216E-03	0.000496972	up	0.614988923	-0.000649382	stable
CMP	5.083E-01	3.40E-20	stable	0.926162875	-1.33E-19	stable
DC	2.775E-01	-0.001748021	stable	0.007817813	-0.003381998	down
Endothelial cells	4.866E-03	0.020961213	up	0.314097158	0.000755639	stable
Eosinophils	2.088E-01	-4.10E-19	stable	0.141181037	-3.33E-19	stable
Epithelial cells	6.874E-01	0.005733776	stable	1.29E-05	-0.021100424	down
Erythrocytes	1.878E-01	0	stable	3.82E-08	2.82E-22	up

iDC	5.340E-01	0.007599046	stable	3.98E-07	0.025146864	up
Keratinocytes	2.892E-01	0.001458944	stable	0.036691843	-0.000454253	down
ly Endothelial cells	9.503E-02	0.007125281	stable	0.690547473	-0.001020907	stable
Memory B-cells	1.466E-01	7.48E-19	stable	0.003912395	-1.96E-18	down
Mesangial cells	3.133E-01	-0.004690843	stable	0.006475941	-0.006156014	down
MPP	6.311E-01	4.70E-20	stable	0.326123481	5.31E-20	stable
MSC	4.367E-07	0.011355511	up	0.134089068	0.001049123	stable
mv Endothelial cells	1.730E-01	0.004656527	stable	0.003543501	-0.010757446	down
Myocytes	3.434E-01	0.000899979	stable	0.018173651	-0.001277488	down
naive B-cells	3.273E-01	-2.05E-19	stable	0.203925716	-2.97E-19	stable
Neutrophils	5.413E-08	-0.001066929	down	0.131387889	-3.85E-20	stable
NK cells	9.350E-01	1.30E-20	stable	0.461670386	1.40E-19	stable
Platelets	9.665E-02	-2.28E-19	stable	0.001135274	-1.97E-19	down
Skeletal muscle	2.447E-01	2.11E-19	stable	0.532222727	4.91E-19	stable
Smooth muscle	9.672E-01	-0.004362417	stable	9.98E-05	0.019338765	up
Tgd cells	7.815E-03	4.64E-19	up	0.16885703	6.57E-19	stable
Tregs	1.259E-01	-0.005459395	stable	0.427446902	3.02E-19	stable

Wilcoxon test was used for comparisons and $p<0.05$ was considered significant.

Table S6. The correlations of CD5L, LCAT and CDC20 with the infiltrations of the immune and stroma cells in HCC.

	Correlations of CD5L with the infiltrations of the immune and stroma cells			
	TCGA-HCC		ICGC-HCC	
	R	*P* value	R	*P* value
aDC	0.298	4.81E-09**	0.250	0.000158**
Adipocytes	0.181	0.00046**	0.314	1.68E-06**
Astrocytes	-0.075	0.151	-0.134	0.0459*
B-cells	0.014	0.789	-0.056	0.404
Basophils	0.026	0.622	-0.052	0.436
CD4+_memory_T-cells	0.130	0.0125*	0.126	0.0607
CD4+_naive_T-cells	0.198	0.000125**	0.002	0.977
CD4+_T-cells	0.041	0.435	0.113	0.0916
CD4+_Tcm	-0.063	0.226	0.154	0.0217*
CD4+_Tem	0.082	0.116	0.090	0.183
CD8+_naive_T-cells	0.206	0.0000627**	0.149	0.0263*
CD8+_T-cells	0.377	5.73E-14**	0.240	0.0003**
CD8+_Tcm	0.400	8.88E-16**	0.401	5.32E-10**
CD8+_Tem	0.219	0.0000201**	0.367	1.57E-08**
cDC	0.415	0**	0.407	2.49E-10**
Chondrocytes	0.050	0.335	-0.105	0.117
Class-switched_memory_B-cells	-0.006	0.913	0.057	0.396
CLP	-0.185	0.000332**	-0.309	0.0000026**
CMP	0.010	0.853	0.175	0.00874**
DC	0.276	6.74E-08**	0.204	0.00215**
Endothelial_cells	0.274	7.95E-08**	0.222	0.000833**
Eosinophils	0.018	0.723	-0.120	0.0745
Epithelial_cells	-0.362	6.01E-13**	-0.340	1.93E-07**
Erythrocytes	-0.009	0.858	0.045	0.503
Fibroblasts	0.066	0.202	0.013	0.85
GMP	0.346	6.82E-12**	0.419	7.09E-11**
Hepatocytes	0.214	0.0000324**	0.437	8.42E-12**
HSC	0.341	1.57E-11**	0.428	2.46E-11**
iDC	0.306	1.84E-09**	0.113	0.0922
Keratinocytes	-0.356	1.61E-12**	-0.310	2.36E-06**
ly_Endothelial_cells	0.263	2.67E-07**	0.221	0.00089**
Macrophages	0.328	9.59E-11**	0.287	0.0000135**
Macrophages_M1	0.294	7.67E-09**	0.228	0.000596**
Macrophages_M2	0.270	1.3E-07**	0.346	1.17E-07**
Mast_cells	-0.023	0.657	-0.027	0.693
Megakaryocytes	0.205	0.0000723**	0.283	0.0000177**
Melanocytes	0.005	0.923	-0.036	0.593
Memory_B-cells	0.109	0.0364	0.009	0.894

MEP	0.063	0.229	-0.091	0.178
Mesangial_cells	-0.088	0.0921	-0.163	0.0147*
Monocytes	0.213	0.0000367**	0.326	6.6E-07**
MPP	-0.083	0.112	0.100	0.135
MSC	0.051	0.332	-0.110	0.102
mv_Endothelial_cells	0.301	3.47E-09**	0.243	0.000256**
Myocytes	-0.251	9.46E-07**	-0.192	0.00395**
naive_B-cells	0.041	0.429	0.125	0.0632
Neurons	-0.160	0.00193**	-0.329	5.07E-07**
Neutrophils	-0.041	0.434	-0.052	0.441
NK_cells	0.020	0.705	0.008	0.908
NKT	-0.164	0.00155**	-0.310	2.28E-06**
Osteoblast	-0.120	0.0206*	-0.235	0.000395**
pDC	0.282	3.41E-08**	0.260	0.0000882**
Pericytes	0.167	0.00125**	0.292	9.24E-06**
Plasma_cells	0.041	0.435	0.130	0.0529
Platelets	-0.016	0.764	-0.048	0.479
Preadipocytes	0.143	0.00584**	0.278	0.0000246**
pro_B-cells	-0.002	0.973	-0.094	0.161
Sebocytes	-0.279	4.4E-08**	-0.403	4.11E-10**
Skeletal_muscle	0.009	0.862	0.140	0.0363*
Smooth_muscle	-0.074	0.156	-0.152	0.0227*
Tgd_cells	-0.035	0.501	-0.148	0.0276*
Th1_cells	0.014	0.795	-0.134	0.0457*
Th2_cells	0.007	0.891	-0.070	0.301
Tregs	0.162	0.0018**	0.187	0.00505**

	Correlations of LCAT with the infiltrations of the immune and stroma cells			
	TCGA-HCC		ICGC-HCC	
	R	*P* value	R	*P* value
aDC	0.014	0.787	-0.088	0.188
Adipocytes	0.379	4.37E-14**	0.503	8.88E-16**
Astrocytes	-0.125	0.0162*	0.005	0.942
B-cells	-0.157	0.00244**	-0.132	0.0494*
Basophils	-0.152	0.00344**	-0.116	0.0843
CD4+_memory_T-cells	-0.161	0.00181**	-0.294	7.96E-06**
CD4+_naive_T-cells	0.074	0.157	0.102	0.128
CD4+_T-cells	-0.048	0.358	-0.074	0.27
CD4+_Tcm	0.077	0.141	0.343	1.53E-07**
CD4+_Tem	-0.01	0.845	-0.036	0.591
CD8+_naive_T-cells	0.203	0.0000822**	0.007	0.919
CD8+_T-cells	0.072	0.167	0.023	0.73
CD8+_Tcm	0.155	0.00277**	0.013	0.849
CD8+_Tem	-0.062	0.236	0.083	0.218
cDC	0.135	0.00925**	0.058	0.387

Chondrocytes	0.026	0.615	-0.069	0.308
Class-switched_memory_B-cells	-0.172	0.000907**	0.009	0.893
CLP	-0.22	0.0000195**	-0.475	6E-14**
CMP	0.166	0.00136**	0.335	3.06E-07**
DC	0.013	0.796	-0.093	0.166
Endothelial_cells	0.319	3.11E-10**	0.1	0.137
Eosinophils	-0.03	0.563	0.12	0.0739
Epithelial_cells	-0.223	0.0000148**	-0.1	0.136
Erythrocytes	0.014	0.789	0.105	0.117
Fibroblasts	0.157	0.00245**	0.105	0.119
GMP	0.194	0.000176**	0.252	0.000139**
Hepatocytes	0.454	0**	0.497	2.44E-15**
HSC	0.394	3.11E-15**	0.281	0.0000202**
iDC	0.096	0.0648	-0.179	0.0073**
Keratinocytes	-0.175	0.0007**	-0.11	0.101
ly_Endothelial_cells	0.356	1.62E-12**	0.174	0.00935**
Macrophages	0.102	0.0488*	-0.124	0.0645
Macrophages_M1	0.023	0.654	-0.104	0.121
Macrophages_M2	0.237	3.97E-06**	0.066	0.328
Mast_cells	-0.223	0.0000148**	-0.323	8.13E-07**
Megakaryocytes	0.279	4.85E-08**	0.25	0.000163**
Melanocytes	-0.071	0.174	-0.015	0.829
Memory_B-cells	-0.047	0.372	-0.143	0.0334*
MEP	0.163	0.00166**	-0.134	0.0461*
Mesangial_cells	-0.11	0.0348*	-0.059	0.379
Monocytes	-0.003	0.951	-0.016	0.815
MPP	0.02	0.702	-0.1	0.136
MSC	0.036	0.492	-0.009	0.899
mv_Endothelial_cells	0.282	3.15E-08**	0.139	0.0375*
Myocytes	-0.065	0.209	0.008	0.901
naive_B-cells	-0.081	0.117	0.088	0.191
Neurons	-0.054	0.302	0.039*	0.559
Neutrophils	0.033	0.531	-0.016	0.811
NK_cells	-0.055	0.292	-0.018	0.789
NKT	-0.205	0.0000698**	-0.086	0.202
Osteoblast	-0.116	0.0261*	-0.206	0.00198**
pDC	0.002	0.973	0.005	0.935
Pericytes	0.213	0.0000352**	0.341	1.76E-07**
Plasma_cells	-0.015	0.774	0.043	0.519
Platelets	-0.042	0.421	-0.054	0.423
Preadipocytes	0.066	0.207	0.055	0.412
pro_B-cells	-0.145	0.00514**	-0.17	0.0112*
Sebocytes	-0.153	0.00318**	-0.187	0.00518**

Skeletal_muscle	0.037	0.48	0.304	3.81E-06**
Smooth_muscle	-0.123	0.0176*	-0.308	2.82E-06**
Tgd_cells	-0.001	0.983	0.006	0.926
Th1_cells	-0.099	0.0573	-0.057	0.397
Th2_cells	-0.315	5.17E-10**	-0.52	0**
Tregs	0.063	0.223	-0.022	0.749

	Correlations of CDC20 with the infiltrations of the immune and stroma cells			
	TCGA-HCC		ICGC-HCC	
	R	*P* value	R	*P* value
aDC	0.134	0.00964**	0.03	0.652
Adipocytes	-0.499	0**	-0.46	4.25E-13**
Astrocytes	0.084	0.107	0.245	0.000226**
B-cells	0.446	0**	0.236	0.000372**
Basophils	0.339	1.98E-11**	0.247	0.000196**
CD4+_memory_T-cells	0.232	0.0000066**	0.199	0.00289**
CD4+_naive_T-cells	0.072	0.167	0.064	0.339
CD4+_T-cells	-0.014	0.789	0.06	0.372
CD4+_Tcm	-0.206	0.0000624**	-0.354	5.66E-08**
CD4+_Tem	-0.131	0.0118*	-0.018	0.792
CD8+_naive_T-cells	-0.077	0.139	-0.157	0.0194*
CD8+_T-cells	0.068	0.19	-0.045	0.503
CD8+_Tcm	-0.018	0.724	-0.124	0.0646
CD8+_Tem	0.155	0.00282**	-0.178	0.00785**
cDC	-0.151	0.00345**	-0.141	0.0357*
Chondrocytes	-0.143	0.00585**	-0.065	0.332
Class-switched_memory_B-cells	0.321	2.29E-10**	0.081	0.23
CLP	0.486	0**	0.493	4.89E-15**
CMP	-0.055	0.294	-0.192	0.00409**
DC	0.108	0.0375*	0.068	0.309
Endothelial_cells	-0.529	0**	-0.245	0.00022**
Eosinophils	0.016	0.754	-0.01	0.88
Epithelial_cells	0.309	1.27E-09**	0.308	2.81E-06**
Erythrocytes	0.113	0.0293*	-0.027	0.691
Fibroblasts	-0.259	4.46E-07**	-0.159	0.0173*
GMP	-0.143	0.00569**	-0.264	0.0000648**
Hepatocytes	-0.56	0**	-0.662	0**
HSC	-0.56	0**	-0.523	0**
iDC	-0.029	0.574	0.112	0.0953
Keratinocytes	0.228	8.85E-06**	0.273	0.0000354**
ly_Endothelial_cells	-0.559	0**	-0.299	5.53E-06**
Macrophages	-0.117	0.0241*	-0.041	0.54
Macrophages_M1	0.133	0.0104*	0.083	0.215
Macrophages_M2	-0.355	1.8E-12**	-0.252	0.000143**

Mast_cells	0.152	0.0033**	0.187	0.00509**
Megakaryocytes	-0.45	0**	-0.493	4.89E-15**
Melanocytes	-0.123	0.0182*	-0.095	0.158
Memory_B-cells	0.157	0.0024**	0.102	0.128
MEP	0.126	0.0151*	0.161	0.0163*
Mesangial_cells	0.211	0.0000425**	0.173	0.00975**
Monocytes	0.148	0.00439**	0.025	0.709
MPP	-0.01	0.849	-0.006	0.932
MSC	-0.023	0.662	0.089	0.185
mv_Endothelial_cells	-0.442	0**	-0.16	0.0167*
Myocytes	-0.055	0.292	-0.101	0.133
naive_B-cells	0.029	0.583	0.015	0.826
Neurons	-0.064	0.221	0.033	0.622
Neutrophils	-0.079	0.131	0.018	0.787
NK_cells	0.067	0.195	0.013	0.848
NKT	0.354	2.06E-12**	0.22	0.000919**
Osteoblast	0.156	0.00267**	0.236	0.000373**
pDC	0.003	0.949	-0.129	0.0538
Pericytes	-0.332	5.1E-11**	-0.421	5.57E-11**
Plasma_cells	0.089	0.0858	-0.116	0.085
Platelets	-0.023	0.653	-0.022	0.744
Preadipocytes	-0.397	2E-15**	-0.368	1.53E-08**
pro_B-cells	0.576	0**	0.477	4.8E-14**
Sebocytes	0.172	0.000899**	0.262	0.0000739**
Skeletal_muscle	-0.118	0.0228*	-0.271	0.0000408**
Smooth_muscle	-0.057	0.273	0.191	0.00425**
Tgd_cells	0.135	0.00902**	-0.042	0.528
Th1_cells	0.446	0**	0.268	0.0000492**
Th2_cells	0.704	0**	0.653	0**
Tregs	-0.196	0.000151**	-0.046	0.49

*, $p < 0.05$; **, $p < 0.01$. CLP, common lymphoid progenitors; ly_endothelial cells, lymphatic endothelial cells. Spearman correlation analysis was used and $p < 0.05$ was considered significant.

Table S7. The prognostic effects of the immune and stroma cells correlated with CD5L,LCAT, and/or CDC20 in HCC.

	TCGA-HCC		ICGC_HCC	
	HR (95%CI)	*P* value	HR (95%CI)	*P* value
Adipocytes	0.79(0.709-0.88)	2.03E-05**	0.771(0.66-0.902)	0.00114**
StromaScore	0.766(0.663-0.886)	0.000312**	0.692(0.586-0.816)	1.30E-05**
Pericytes	0.872(0.8-0.95)	0.00171**	0.897(0.79-1.02)	0.0921
Th2_cells	1.13(1.04-1.23)	0.00279**	1.86(1.36-2.56)	0.000125**
Megakaryocytes	0.819(0.712-0.941)	0.00491**	0.757(0.562-1.02)	0.0676
pro_B-cells	1.12(1.03-1.21)	0.00561**	1.32(1.12-1.56)	0.00125**
MicroenvironmentScore	0.743(0.599-0.921)	0.00677**	0.647(0.506-0.827)	0.000515**
ly_Endothelial_cells	0.9(0.825-0.982)	0.0172*	0.847(0.728-0.986)	0.0323*
Macrophages_M2	0.85(0.742-0.973)	0.0181*	0.918(0.755-1.12)	0.392
HSC	0.915(0.846-0.988)	0.0238*	0.88(0.758-1.02)	0.093
Monocytes	1.09(1.01-1.19)	0.0367*	0.944(0.823-1.08)	0.413
CLP	1.15(1.01-1.31)	0.0381*	1.61(1.19-2.18)	0.00212**
Osteoblast	1.1(0.998-1.22)	0.0536	1.17(1.02-1.34)	0.0208*
CD8+_Tcm	0.932(0.865-1)	0.0615	0.865(0.754-0.992)	0.0386*
Preadipocytes	0.938(0.877-1)	0.0647	0.995(0.878-1.13)	0.944
CD8+_T-cells	0.934(0.863-1.01)	0.0893	0.936(0.819-1.07)	0.331
Endothelial_cells	0.918(0.83-1.01)	0.0923	0.898(0.785-1.03)	0.114
pDC	0.923(0.841-1.01)	0.0943	1.04(0.882-1.22)	0.654
Th1_cells	1.1(0.98-1.23)	0.106	1.09(0.911-1.31)	0.344
Tregs	0.93(0.847-1.02)	0.125	0.888(0.674-1.17)	0.397
cDC	0.941(0.856-1.03)	0.207	0.792(0.634-0.989)	0.0393*
Keratinocytes	1.08(0.95-1.23)	0.241	1.1(0.923-1.32)	0.283
Hepatocytes	0.926(0.812-1.05)	0.245	0.956(0.824-1.11)	0.553
mv_Endothelial_cells	0.945(0.853-1.05)	0.278	0.898(0.775-1.04)	0.15
CD4+_Tcm	0.935(0.828-1.06)	0.28	0.717(0.608-0.846)	7.78E-05**
CD8+_Tem	0.937(0.827-1.06)	0.3	0.909(0.786-1.05)	0.201
Mast_cells	1.06(0.939-1.19)	0.358	1.14(0.93-1.39)	0.212
Macrophages_M1	1.04(0.952-1.14)	0.372	0.985(0.855-1.13)	0.829
NKT	1.04(0.951-1.13)	0.405	1.07(0.929-1.23)	0.358
Epithelial_cells	1.06(0.922-1.21)	0.426	1.08(0.942-1.25)	0.263
B-cells	1.03(0.956-1.11)	0.454	0.989(0.871-1.12)	0.859
Basophils	0.974(0.907-1.05)	0.479	1.07(0.954-1.21)	0.244
CMP	0.942(0.792-1.12)	0.496	0.76(0.557-1.04)	0.0823
Mesangial_cells	1.03(0.939-1.13)	0.518	0.939(0.825-1.07)	0.341
CD4+_memory_T-cells	1.03(0.927-1.15)	0.562	1.01(0.859-1.18)	0.937
DC	0.977(0.883-1.08)	0.647	0.931(0.798-1.09)	0.364
Macrophages	0.978(0.881-1.09)	0.681	0.944(0.823-1.08)	0.415

Myocytes	0.974(0.858-1.11)	0.682	1.05(0.851-1.31)	0.629
GMP	0.982(0.889-1.08)	0.715	0.849(0.689-1.05)	0.126
Neurons	0.961(0.756-1.22)	0.743	0.829(0.539-1.27)	0.392
aDC	1(0.943-1.07)	0.894	0.966(0.869-1.07)	0.518
ImmuneScore	0.995(0.894-1.11)	0.933	0.902(0.762-1.07)	0.232
Sebocytes	1(0.871-1.15)	0.969	1.1(0.883-1.36)	0.404
CD8+_naive_T-cells	0.888(0.782-1.01)	0.068	1.04(0.786-1.37)	0.787
Smooth_muscle	1.22(0.989-1.5)	0.0628	1.4(0.896-2.2)	0.139
Fibroblasts	0.945(0.884-1.01)	0.101	0.795(0.692-0.914)	0.00129**
MEP	1.17(0.982-1.4)	0.0784	1.71(1.13-2.58)	0.0119*
Skeletal_muscle	0.979(0.814-1.18)	0.825	0.863(0.608-1.23)	0.412

*, *p* < 0.05; **, *p* < 0.01. CLP, common lymphoid progenitors; ly_endothelial cells, lymphatic endothelial cells.Multi-variable cox regression analysis was used and the age-gender-stage corrected prognostic effects of the cells and scores were evaluated. The cells/scores with significant prognostic effects (*p* < 0.05) consistently in the two datasets were considered significant. The lines filled with green color indicated favorable prognostic effects while the ones filled with yellow color indicated unfavorable prognostic effects.

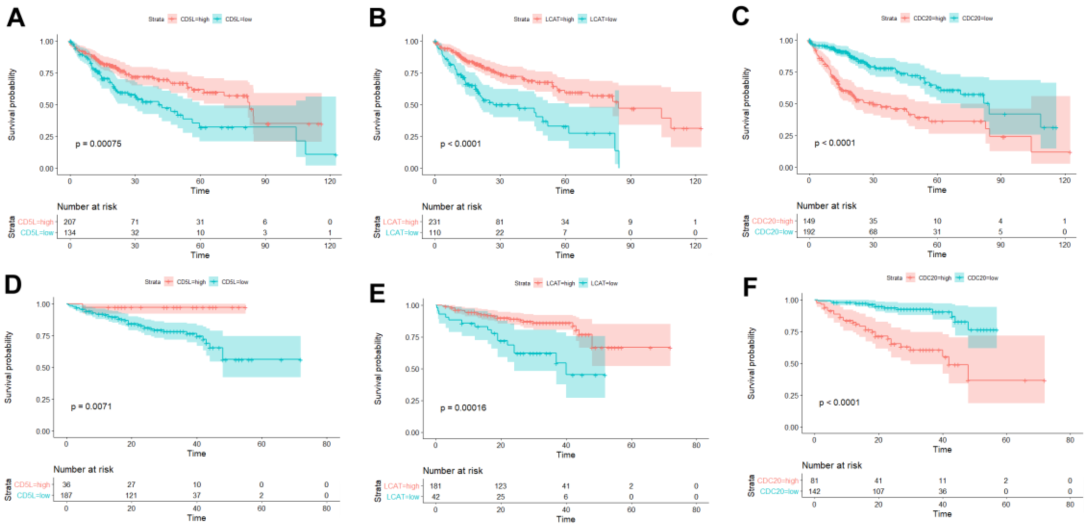

Figure S1. Prognostic effects of CD5L, LCAT and CDC20 on HCC OS. (A-B) Significant favorable prognostic effects of CD5L and LCAT on HCC OS in TCGA-HCC dataset. (C) Significant unfavorable prognostic effects of CDC20 on HCC OS in TCGA-HCC dataset. (D-E) Significant favorable prognostic effects of CD5L and LCAT on HCC OS in ICGC-HCC dataset. (F) Significant unfavorable prognostic effects of CDC20 on HCC OS in ICGC-HCC dataset. OS, overall survival; HCC, hepatocellular carcinoma. Kaplan-Meier survival analysis was used and p < 0.05 was considered significant.

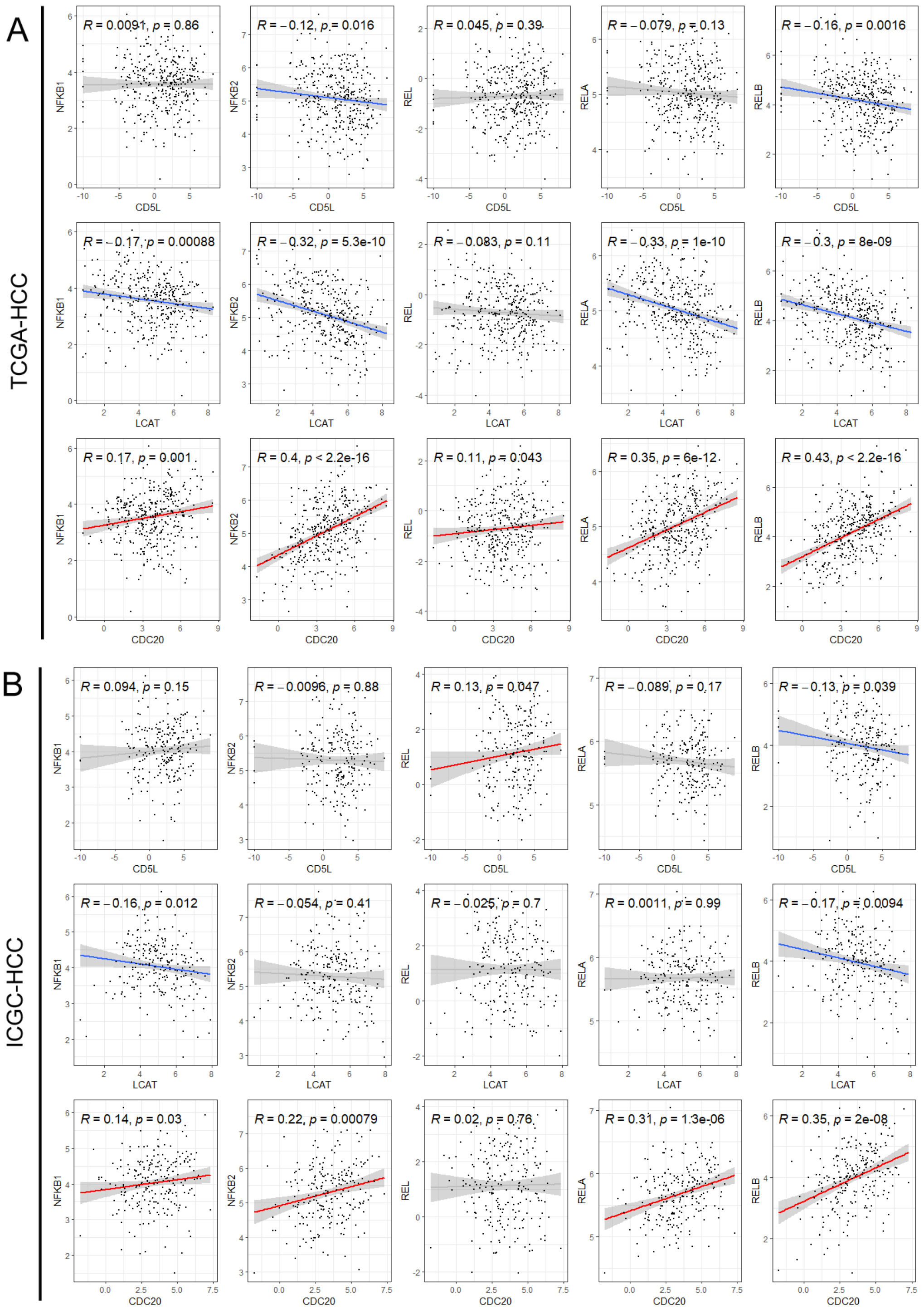

Figure S2. The correlations of CD5L, LCAT and CDC20 expressions with NK-κB associated genes. (A) The correlations of CD5L, LCAT and CDC20 expressions with NFKB1, NFKB2, REL, RELA, and RELB expressions in TCGA-HCC samples. (B) The correlations of CD5L, LCAT and CDC20 expressions with NFKB1, NFKB2, REL, RELA, and RELB expressions in ICGC-HCC samples. Spearman correlation analysis was used and $p < 0.05$ was considered significant.

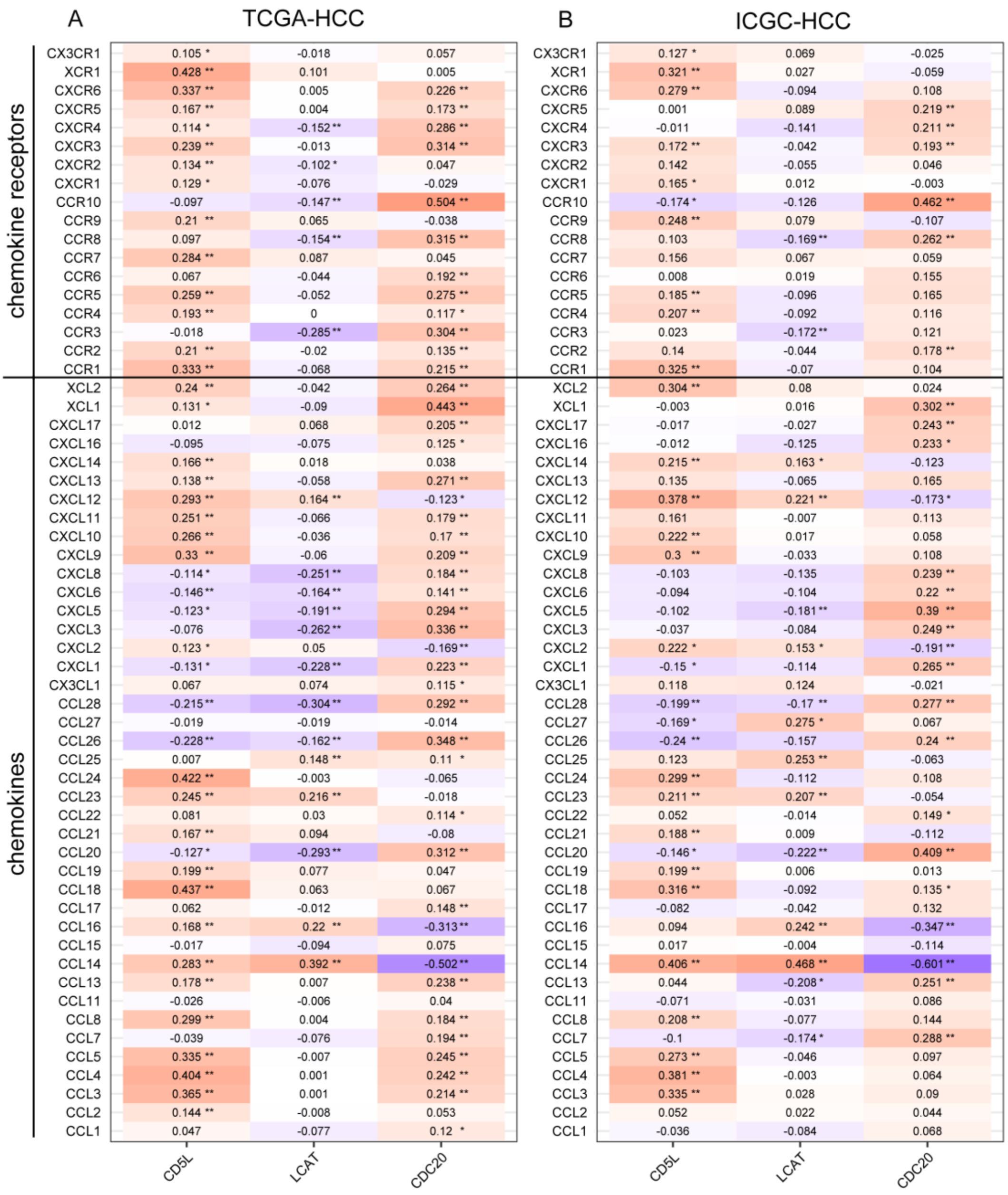

Figure S3. The correlations of CD5L, LCAT, and CDC20 with chemokines and chemokine receptors in HCC. (A) The correlations of CD5L, LCAT, and CDC20 with chemokines and chemokine receptors in TCGA-HCC samples. (B) The correlations of CD5L, LCAT, and CDC20 with chemokines and chemokine receptors in ICGC-HCC samples. Spearman correlation analysis was used and $p < 0.05$ was considered significant. The correlation coefficients were shown and the significances was indicated (*, $p < 0.05$; **, $p < 0.01$).

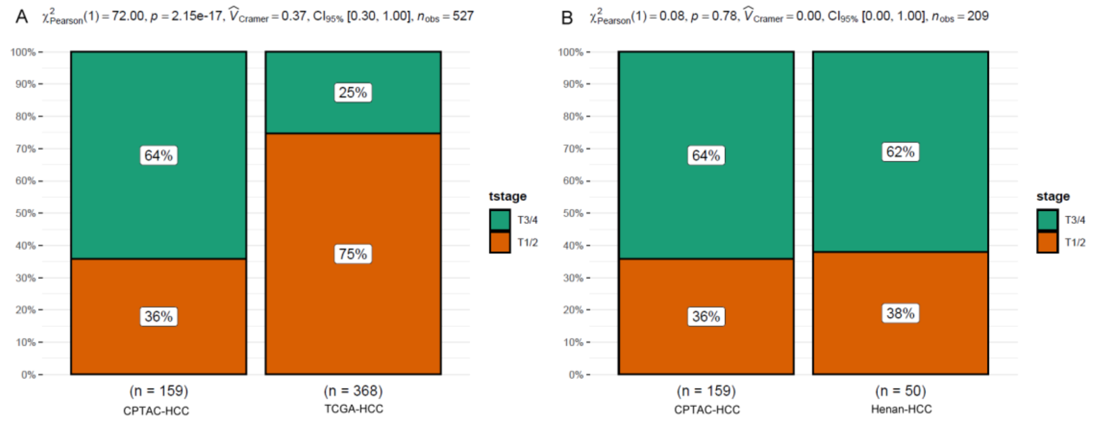

Figure S4. Tumor stage proportion comparisons between different HCC datasets. (A) Significant higher T3/T4 proportion samples in CPTAC dataset than TCGA-HCC dataset. (B) There was no significant tumor stage difference between CPTAC-HCC dataset and the HCC samples from Henan People's Hospital. Chi-square test was used for the comparisons and p < 0.05 was considered significant.

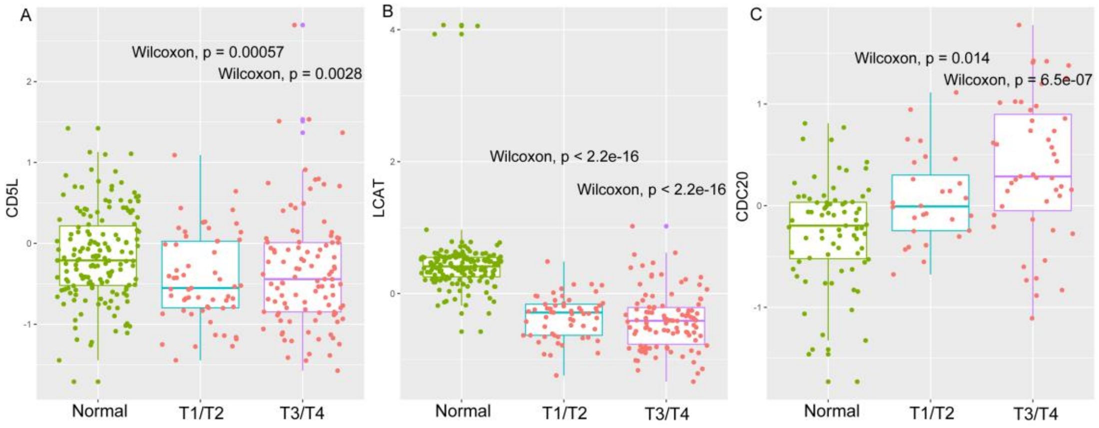

Figure S5. CD5L, LCAT and CDC20 expression comparisons between CPTAC-HCC samples of different stages and normal livers. (A) Significant lower expression of CD5L in HCC samples of different stages than normal liver tissues. (B) Significant lower expression of LCAT in HCC samples of different stages than normal liver tissues. (C) Significant higher expression of CDC20 in HCC samples of different stages than normal liver tissues. Wilcoxon test was used and p < 0.05 was considered significant.

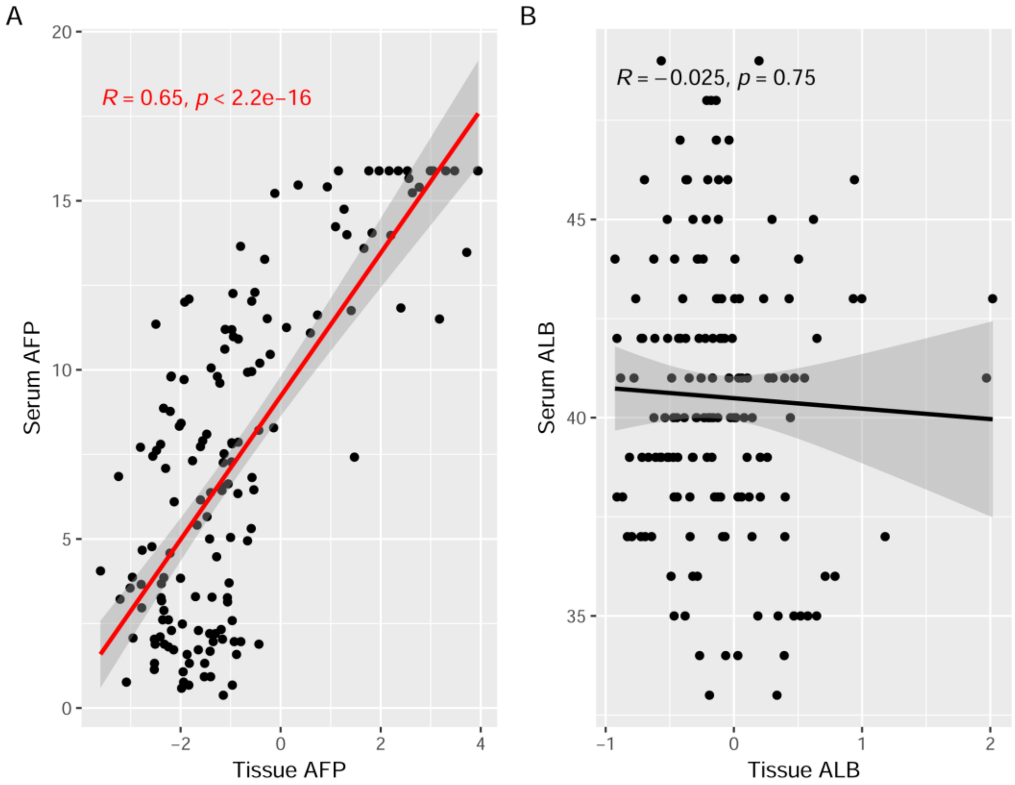

Figure S6. Correlations of tissue AFP and ALB with serum AFP and ALB in HCC. (A) Tissue AFP level in HCC was significant correlated with serum AFP (ng/ml). (B) There was no significant correlation between tissue ALB and serum ALB level (g/l) in HCC. For (A) the serum AFP (ng/ml) was log2(x) transformed. Spearman correlation analysis was used and p<0.05 was considered significant.

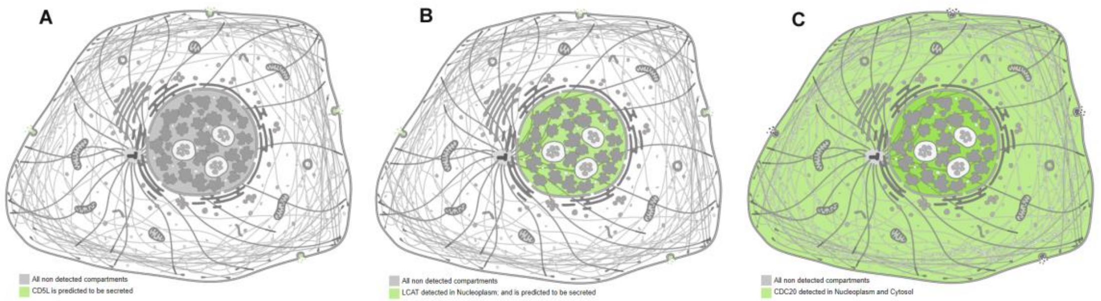

Figure S7. Subcellular location of CD5L, LCAT and CDC20 in human protein atlas (HPA) (https://www.proteinatlas.org/). (A-C) The subcellular locations of CD5L, LCAT and CDC20, respectively.

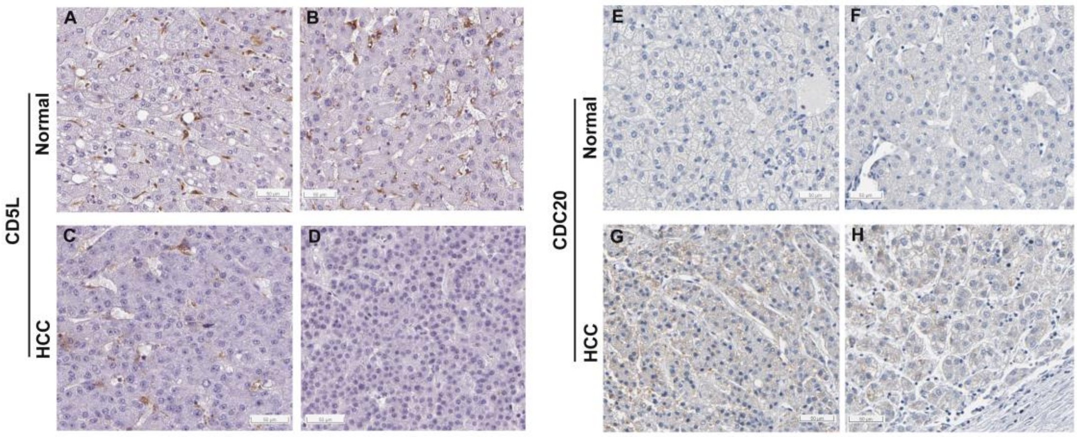

Figure S8. Immunohistochemistry staining of CD5L and CDC20 in HCC and liver tissues. (A-B) Positive staining of CD5L in liver stroma. (C) Positive staining of CD5L in HCC stroma. (D) negative staining of CD5L in HCC stroma. (E-F) Negative staining of CDC20 in liver tissues. (G-H) Positive staining of CDC20 in HCC tissues. The IHC data were from human protein atlas (HPA, https://www.proteinatlas.org/) and the corresponding antibodies were HPA065686 (CD5L) and CAB004525 (CDC20).
